# Supplementary material for: Homogentisic acid-derived pigment as a biocompatible label for optoacoustic imaging of macrophages
Source: Nat Commun. 2019 Nov 7;10:5056. doi: 10.1038/s41467-019-13041-4 (PMC6838096; doi:10.1038/s41467-019-13041-4)
Supplement: Supplementary file 1 — Supplementary Information [file 41467_2019_13041_MOESM1_ESM.pdf]

Supplementary Information

Homogentisic acid-derived pigment as a biocompatible label for optoacoustic imaging of macrophages

Ina Weidenfeld[a], Christian Zakian[a,b], Peter Duewell[c], Andriy Chmyrov[a,d], Uwe Klemm[a] Juan Aguirre[a,b], Vasilis Ntziachristos[a,b,d] and Andre C. Stiel[a,*]

a) Institute of Biological and Medical Imaging (IBMI), Helmholtz Zentrum München, Neuherberg, Germany

b) Chair of Biological Imaging, Technische Universität München, Munich, Germany

c) Institute of Innate Immunity, University of Bonn, Bonn, Germany

d) Center for Translational Cancer Research (TranslaTUM), Technische Universität München, Munich, Germany

Supplemental Notes 1: Cellular pathways for HDP formation in macrophages

It is conceivable that both extracellular HGA monomers and HDP intermediaries, but also fully polymerized HDP, can be endocytosed via fluid phase uptake.

To test the possibility of fluid phase uptake of already polymerized HDP as a source for pigmentation, we treated Ana-1 cells with the macropinocytosis inhibitor DMA (5-(N,N-dimethyl)-amiloride hydrochloride [1]), followed by incubation with HGA or Alexa750 as a control (Suppl. Figure 1a). While macropinocytosis was successfully blocked, shown by the cellular exclusion of Alexa750, Ana-1 cells accumulated the pigment only at a slightly lower level than in the absence of the inhibitor, suggesting ongoing HGA diffusion followed by intracellular polymerization.

To further examine whether HDP polymerized extracellularly could contribute to pigmentation, we incubated 0.3 mM HGA in cell-free growth media for 72h of pre-polymerization before exposing it to Ana-1 cells for 24h of uptake. In parallel, control cells were co-cultured with 0.3 mM HGA for 96h. Suppl. Figure 1b shows limited cellular uptake of cell-free polymers: the coloration and OA signal were much lower than for cells co-cultured with HGA for 96h. This result suggests that HDP formation occurs primarily intracellularly after uptake. This is furthermore supported by the repeated observation that a 96h incubation with pre-polymerized pigment leads to high cell mortality among the exposed population. Interestingly, when Ana-1 cells were repeatedly supplemented with HGA while undergoing media changes to prevent extracellular polymer formation, cells show a decreased level of pigmentation compared to continuous co-incubation with HGA as described above (Suppl. Figure 1c).

To probe the potential role of the intracellular environment in promoting HGA polymerization, we followed polymerization kinetics in growth media at stable neutral pH and over 96h in the presence and absence of Ana-1 cells as well as in presence of cellular debris by absorbance spectroscopy (Suppl. Figure 2). In all 3 cases, the spectra show features consistent with HGA (290 nm) as well as with 1,4- benzoquinone-2-acetic acid (BQA; 250 nm), an auto-oxidation product of HGA that preferentially forms under strongly acidic or alkaline conditions [2,3]. HGA absorbance is precisely detected at 0h, immediately after the single-dose supplementation with 0.3 mM HGA. With the onset of HGA oxidation to BQA throughout 96h, the latter signal increases at 250 nm with the strongest absorbance measured in the presence of intact cells. BQA has a high reactivity towards compounds carrying free amine and sulfhydryl groups which may lead to their incorporation during polymerization [2,3]. These BQA intermediaries are detected at 340 and 470/490 nm with an increase in absorbance at 340 nm over time. Importantly, we also notice an increase in absorbance starting at 600 nm and persisting to 900 nm only in the presence of intact cells. We speculate that this absorbance peak corresponds to the HDP responsible for the OA signals we detect in cells at 630 nm (RSOM) and > 680 nm (MSOT). We hypothesize, therefore, that HDP forms from HGA via BQA. Consistent with this idea, HGA and BQA are known to form a redox system with a highly positive redox potential of BQA [4] which consequently leads to the formation of homo-/heterodimers and small oxidized polymers thereof [5].

Supplemental Notes 2: Intracellular localization of HDP

To determine the intracellular localization of HDP in primary macrophages more precisely, we conducted bright field, fluorescence and high-resolution OA microscopy. Cells growing on poly-L-lysine coated glass cover slips or glass bottom dishes were incubated with 0.5 mM HGA for 96h, briefly fixed with 4% PFA, then stained for melanin using Schmorl's stain [6] or immunostained with fluorescently labeled antibodies against lysosomal-associated membrane protein Lamp1, a widely accepted lysosomal marker. HDP was successfully identified by bright field microscopy and found localized within intracellular vesicles distributed in the cytoplasm with partial perinuclear accumulation (Figure 2a). This was furthermore verified by blue

Schmorl's staining. By taking advantage of the OA properties of HDP, we resolved single pigmented vesicles and identified them as late endosomes/lysosomes based on co-localization with Lamp1 fluorescence (Figure 2b). This result further supports our theory that HGA monomers as well as BQA intermediaries first cross the membrane by direct diffusion or transporter-mediated translocation to then accumulate in endolysosomal organelles where high acidity might facilitate polymerization and HDP formation.

Supplemental Notes 3: OA properties and viability of macrophages labeled with HDP

We evaluated signal retention by culturing Ana-1 cells first in medium containing HGA or synthetic melanin so that they attained similar levels of pigmentation, and then maintaining them in label-free medium. We observe similar behavior for both labels concerning OA signal retention with both systems returning to cellular background level *in vitro* after 24h of depletion (Suppl. Figure 4a). In parallel, OD_{700nm} measurements of growth media within the first 48h reveal an almost linear increase in signal with values reaching saturation at 48h – 72h (Suppl. Figure 4c). The overall level of secreted HDP herein is insignificant and 25% lower than that of secreted synthetic melanin. With cell viability routinely monitored during the course of HGA exposure, it is equally important to assess this factor after depletion of the supplement with regard to longitudinal *in vivo* application of pre-labeled cells. Synthetic melanin exposure takes a toll on cells to such an extent that viability drops below 75% even after 72h of recuperation in label-free growth media. As for HGA-treatment, we observe a significantly smaller effect on cell viability with values at 89% (Suppl. Figure 4b).

Supplemental Notes 4: Verification of macrophage functionality after HDP-labeling

The morphological changes that accompany the cells during transformation are shown in Suppl. Figure 5a. Activated cells present themselves with elongated spindle-shaped cell bodies in comparison to their more star-shaped appearance pre LPS treatment and rounded form before differentiation [7]. This is observed in untreated as well as HGA-pigmented cells. For the cells which have undergone tedious phagocytosis of synthetic melanin, an activated macrophage phenotype was not as apparent compared to HGA-treated or untreated cells, with many cells starting to develop apoptotic vesicles. The assessment of cell viability after cell detachment and repeated PBS washes resulted in 86% viable cells for HGA, 93% for untreated and only 40% for synthetic melanin-supplemented cells rendering the latter impractical for *in vivo* application studies.

A salient features of macrophage function is the presentation of antigen to T-cells (reviewed in [8]). Taking place at close proximity, we demonstrate the lack of intercellular transfer of HGA-derived pigment from macrophages to another type of cell that might be recruited by the presence of IFN-γ and LPS (Suppl. Information and Suppl. Figure 9). To test this, Jurkat cells (T lymphocytes cell line) were co-cultured with HDP-labeled primary macrophages for 24h in vitro. While macrophages remain firmly attached to the surface of cell culture dishes, T-cells are in suspension allowing easy separation of the two cell types after incubation. As a result, no visible pigment was detected in Jurkat cells whereas macrophages remained pigmented after 24h (Suppl. Figure 9).

Furthermore, we tested other tissue-derived mammalian cell lines for their ability to produce pigment in the presence of HGA. Neither Hela -, HEK -, nor Jurkat cells showed intracellular pigmentation upon HGA induction. It is conceivable that the lack of pigmentation is caused by the presence of homogentisate 1,2 dioxygenase, the enzyme responsible for HGA breakdown during tyrosine catabolism in healthy cells [9]. While the enzyme is expressed in human fetal - and adult kidney, explaining the absence of pigment formation in HEK cells, future experiments are needed to elucidate other tissue-specific expression patterns.

1. Sarkar, K., Kruhlak, M. J., Erlandsen, S. L. & Shaw, S. Selective inhibition by rottlerin of macropinocytosis in monocyte-derived dendritic cells. *Immunology* **116**, 513–24 (2005).
2. Stoner, R. & Blivaiss, B. B. Reaction of quinone of homogentisic acid with biological amines. *Arthritis Rheum.* **10**, 53–60 (1967).
3. Lustberg, T. J., Schulman, J. D. & Seegmiller, J. E. The preparation and identification of various adducts of oxidized homogentisic acid and the development of a new sensitive colorimetric assay for homogentisic acid. *Clin. Chim. Acta* **35**, 325–333 (1971).
4. R, C., Forbes, H. A. W., Glynn, L. E. & Stanier, W. M. Observations on the oxidation of homogentisic acid in urine. *Biochem. J.* **50**, 274–8 (1951).
5. Turick, C. E., Knox, A. S., Becnel, J. M., Ekechukwu, A. A. & Milliken, C. E. Properties and Function of Pyomelanin. *Biopolymers* 449–472 (2010). doi:10.5772/10273
6. JD, B. & A, S. *Theory and practice of histological techniques*. (Churchill Livingstone, New York, 1955).
7. Zajac, E. *et al.* Angiogenic capacity of M1- and M2-polarized macrophages is determined by the levels of TIMP-1 complexed with their secreted proMMP-9. *Blood* **122**, 4054–4067 (2013).
8. Biswas, S. K. & Mantovani, A. Macrophage plasticity and interaction with lymphocyte subsets: Cancer as a paradigm. *Nat. Immunol.* **11**, 889–896 (2010).
9. Laschi, M. *et al.* Homogentisate 1,2 dioxygenase is expressed in human osteoarticular cells: Implications in alkaptonuria. *J. Cell. Physiol.* **227**, 3254–3257 (2012).

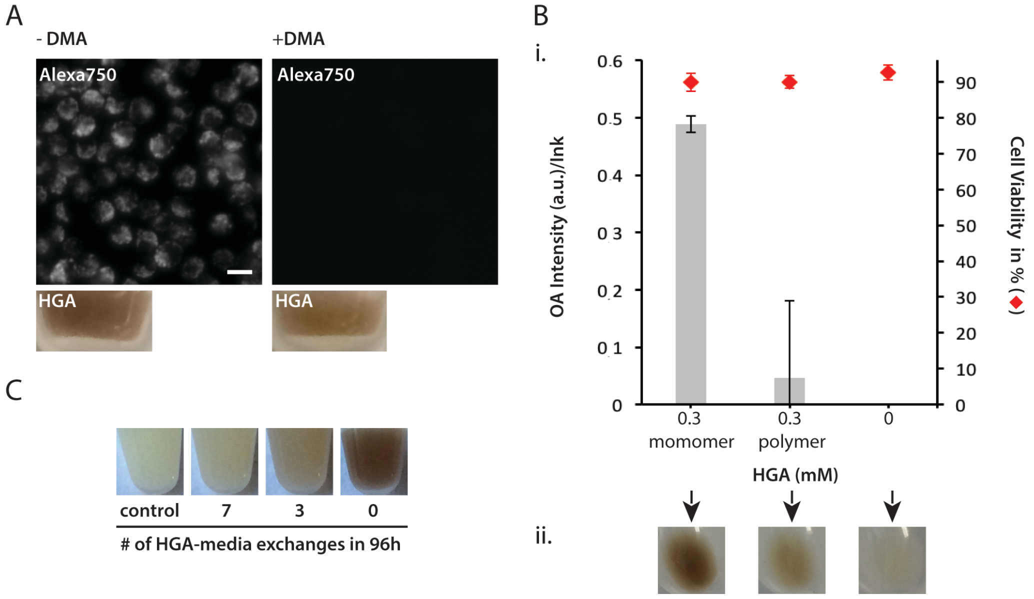

Suppl. Figure 1. HGA-mediated pigmentation occurs *in vivo* and *in situ*. (a) Ana-1 macrophages are treated with or without the macropinocytosis inhibitor DMA, (5-N,N-dimethyl)-amiloride hydrochloride, as well as Alexa 750 or HGA (0.3 mM). While DMA fully blocked the fluid phase uptake of Alexa750 (fluorescence images, *top*), HGA-pigmentation still occurs suggesting intracellular formation of the pigment after membrane diffusion (cell pellets, *bottom*). Scale bar is 10 µm. (b) OA signal intensity and cell viability (i) are determined after cells are treated with 0.3 mM fresh HGA monomer for 96h compared to 24h of pre-polymerized HGA (0.3 mM HGA in cell-free media for 72h prior). Error bars, mean ± SD. Intracellular pigmentation levels of the corresponding cells are shown in (ii). Extended exposure to pre-polymerized HGA is extremely cytotoxic (data not shown). (c) Ana-1 cells are repeatedly supplemented with HGA while media is exchanged to prevent extracellular formation of polymers. Source data are provided as a Source Data file.

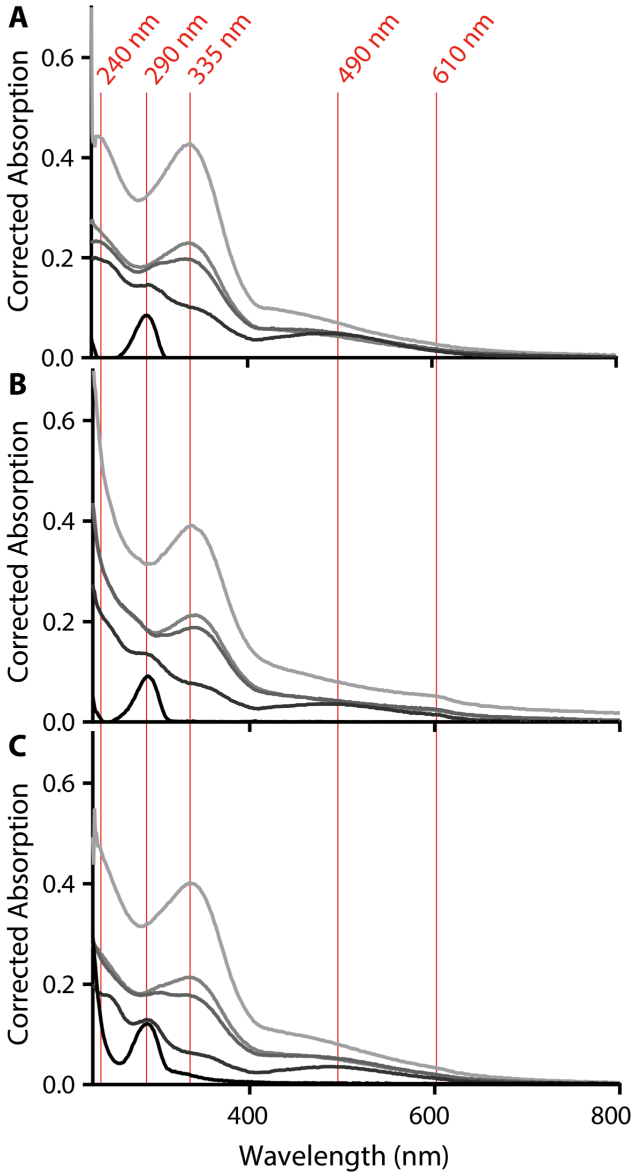

Suppl. Figure 2. Absorbance spectroscopy of HDP formation kinetics in (A) absence and (B) presence of cells as well as (C) with cellular debris. Absorption spectra of the samples taken at 0 (*black*), 24, 48, 72 and 96 h are depicted as shades of gray. HGA is measured at 290 nm, BQA at 240/250 nm and BQA-intermediaries at 340 and 470/490 nm. Only in the presence of cells (B) do we observe increase in absorbance at 240/250 nm (BQA) and at ~600 – 800 nm indicating the production of HDP which we detect with different OA methods (MSOT 680 - 900 nm; RSOM at 650 nm and OA microscopy at 592 nm). RPMI media of each corresponding time point and for each experimental setup (A, B and C) was used as reference and subtracted.

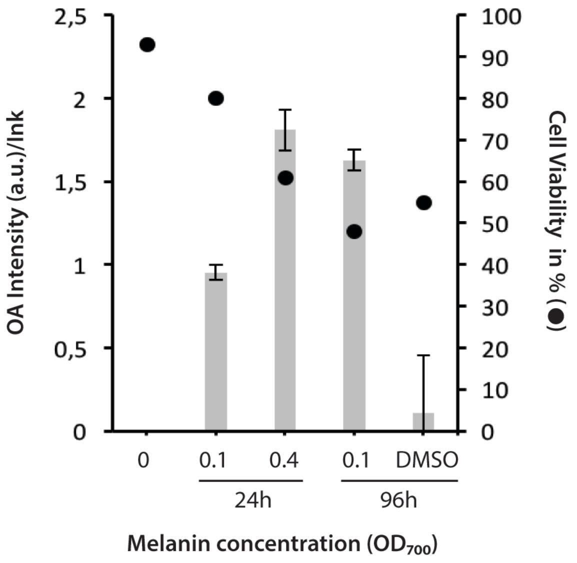

Suppl. Figure 3. OA properties of solubilized synthetic melanin *in vitro*. Macrophages are treated with synthetic melanin, solubilized in DMSO, at an OD_{700nm} of 0.1 and 0.4 for 24h and 96h (final DMSO in growth media <1%). Cells demonstrate strong OA signal (grey columns) accompanied by a strong decrease in viability (black dots). Error bars, mean ± SD. Source data are provided as a Source Data file.

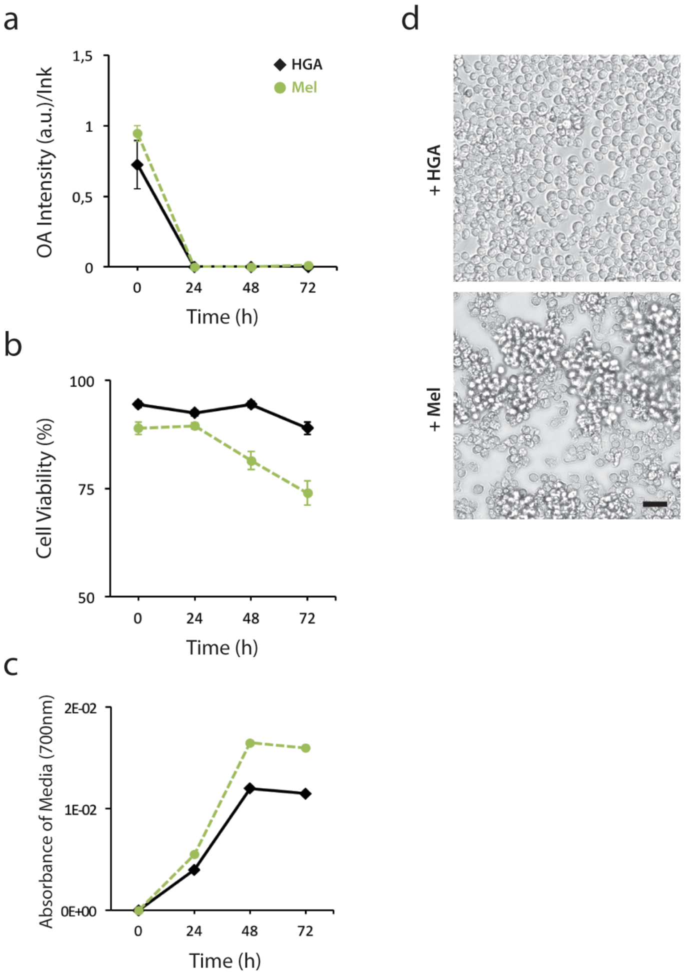

Suppl. Figure 4. Signal retention and cytotoxicity of HDP vs. synthetic eumelanin in macrophages after HGA depletion. (a) OA signal retention and cell viability (b) of Ana-1 cells measured up to 72h after depletion of either HGA (black) or synthetic eumelanin (green) from growth media. (c) Absorbance measurements of growth media at OD_{700nm} for 72h after depletion for the samples shown in a and b. (d) Bright field microscopic images of morphological cell alterations 24h post synthetic melanin exposure as seen in comparison to HGA treatment. Scale bar 30 µm. Source data are provided as a Source Data file.

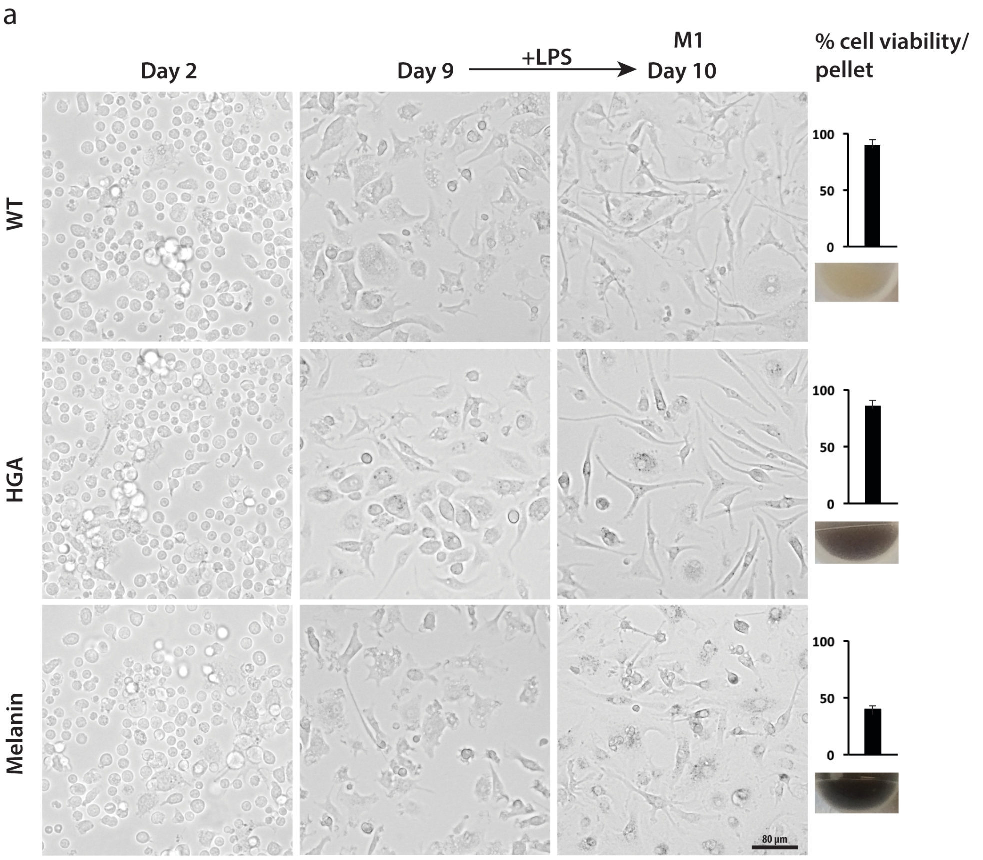

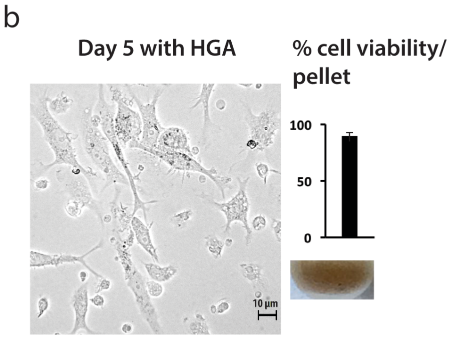

Suppl. Figure 5. Assessment of primary macrophage plasticity after HDP-labeling. (a) Timeline documenting the morphological changes occurring in primary macrophages during differentiation and M1-activation in unlabeled (WT) cells (*top*) and during HGA-labeling (*middle*) or synthetic melanin (*bottom*) treatment. 0.5 mM HGA is added on Day 5 and melanin with OD_{700nm} 0.1 is added on Day 9, both leading to visible pigmentation of the cells on Day 10 as seen in bright field and after cell pelleting. LPS for activation/polarization is added to all samples and for the last 24h before cell collection. M1 cell body elongation occurs in WT and HGA-labeled cells while high cell viability is preserved. Synthetic melanin treatment leads to poorer cell polarization and low viability. Scale bar is 80 µm. (b) HDP-labeling starting as early as Day 2 until Day 5 of differentiation also leads to intracellular pigmentation without compromising viability. Scale bar is 10 µm.

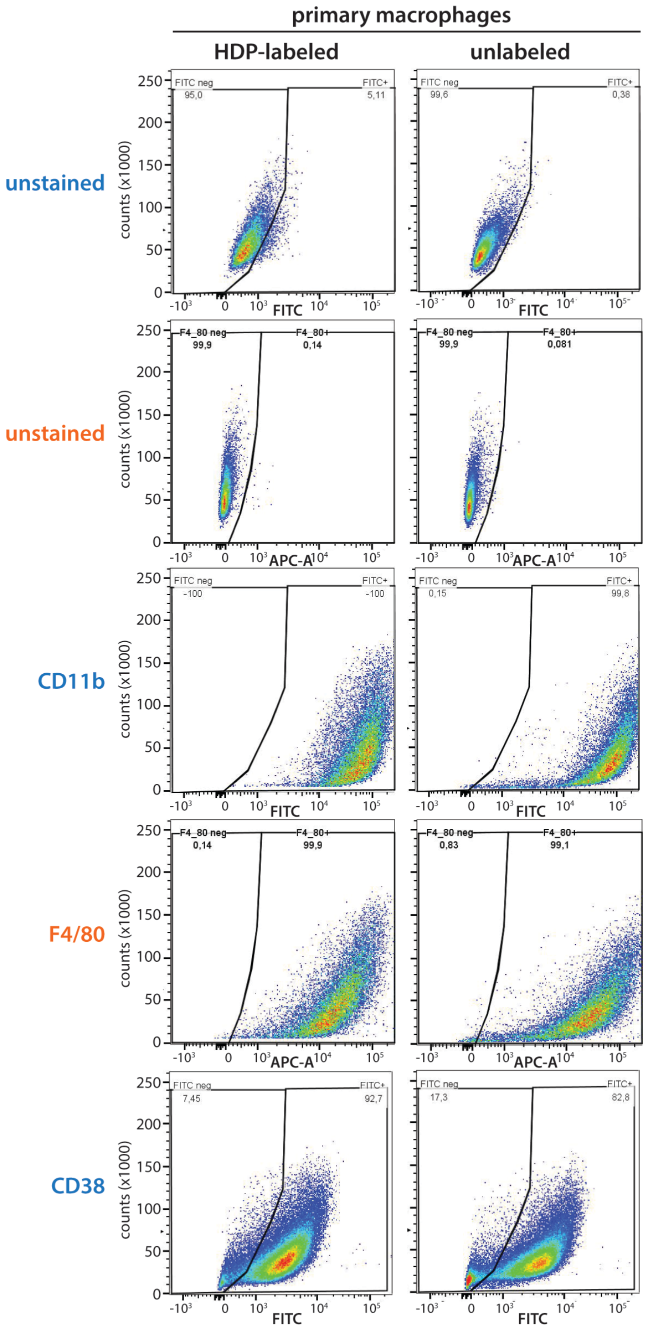

Suppl. Figure 6. Assessment of primary macrophage functionality after HDP-labeling. Density dot plots of flow cytometry analysis show the efficient differentiation of more than 90% of bone marrow derived cells (anti-CD11b) to primary macrophages (M0; anti-F4/80) after 8 days of differentiation and in the presence or absence of HGA for days 5-8. HDP also does not perturb the polarization to activated proinflammatory macrophages after LPS exposure as verified by CD38 surface marker expression (*bottom row*).

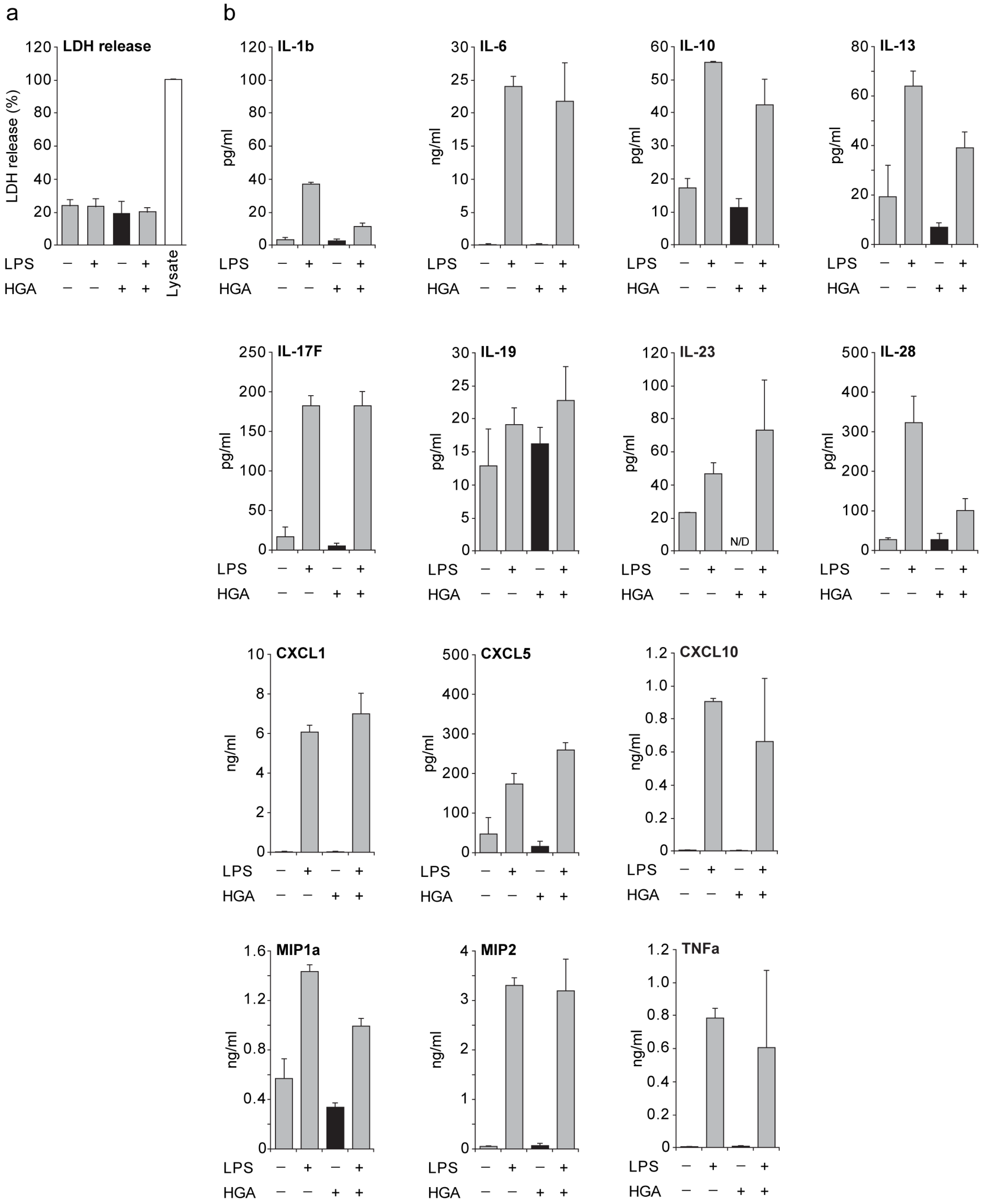

Suppl. Figure 7. HDP-labeling does not affect cellular function and viability of primary macrophages. Cells were differentiated and treated with or without 0.5 mM HGA for 5 days, followed by 3h of 200 ng/ml LPS, or left untreated. (a) Viability was assessed by lactate dehydrogenase (LDH) release in cell culture supernatants. Whole cell lysate was used as positive control (steady-state). (b) Cytokine and chemokine secretion of primary macrophages was assessed in supernatants, using a multiplex cytokine array. Data as mean +SD of n=3 biological replicates. Source data are provided as a Source Data file.

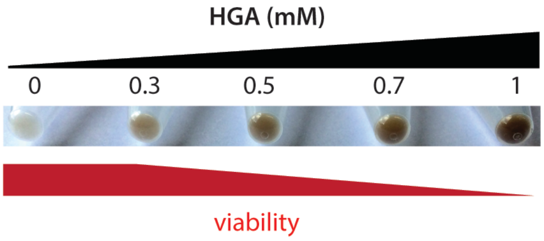

Suppl. Figure 8. HDP formation in mouse embryonic stem cells. mES cells are treated with different concentrations of HGA (0, 0.3, 0.5, 0.7 and 1 mM) for 96h. Increasing levels of intracellular pigmentation occur as a function of concentration. Cell viability does not differ between 0 and 0.3 mM of HGA, however declines rapidly from 0.5 – 1 mM.

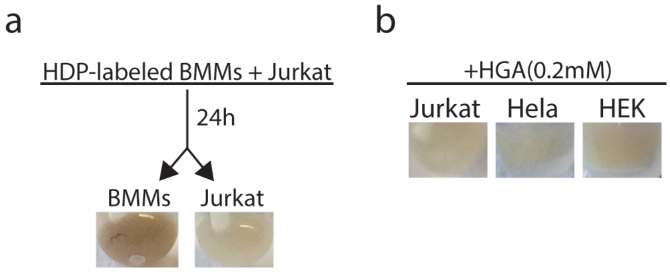

Suppl. Figure 9. Macrophage-specific labeling. (a) Testing intercellular transfer of HDP. HDP-labeled proinflammatory (M1) bone marrow macrophages (BMMs) as prepared for Suppl. Figure 4a, Day 10, are mixed with T-cells for 24h, separated due to their physical properties and visually analyzed for pigmentation. No transfer of pigment or decrease in cell viability is observed. (b) Jurkat -, HeLa - and HEK cells are treated with 0.2 mM HGA for up to 21 days without any visible formation of pigment or decline in cell viability. Concentrations above 0.3 mM HGA lead to high cytotoxicity in these cell lines.

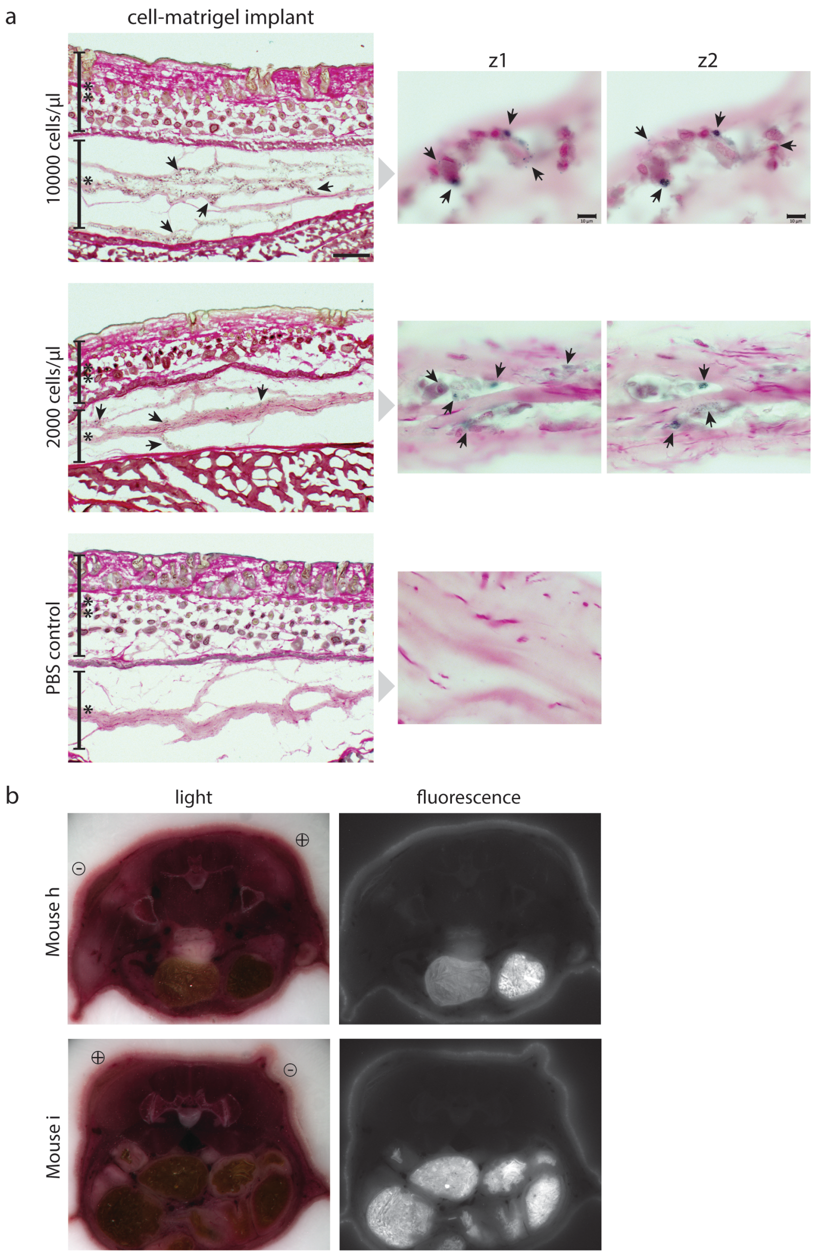

Suppl. Figure 10. Histological overview of cell-matrigel implantations and whole mouse cross sections. (a) Histological tissue sections stained with Schmorl showing s.c. matrigel implants with defined numbers of HDP-labeled primary macrophages mixed into the gel before implantation. The increase in dark contrast from top to bottom (10000 cells/µl, 2000 cells/µl and PBS) is clearly visible. Z scans of 200nm step size were acquired with z1 and z2 chosen to be 2-5 µm apart. The HDP-filled cells are indicated with arrows. Scale bars are 200µm and 10 µm. (b) Whole body cross sections of cryopreserved O.C.T-embedded mice 'h' and 'i' from **Figure 4**. imaged with light and fluorescence (RFP filter). IFNγ-positive and –negative matrigel implants are indicated by (+) and (-).

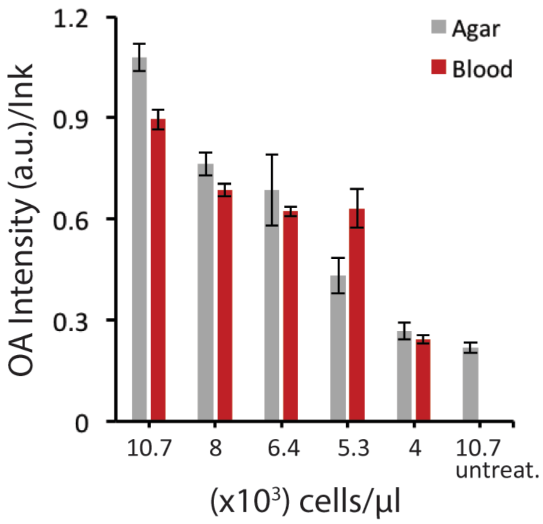

Suppl. Figure 11. In vitro quantification of HDP-laden cells by MSOT. Normalized OA signal intensities are shown as a function of cell number. Decreasing numbers of HDP-labeled primary macrophages were measured while inside blood-agar phantoms vs PBS-agar phantoms to determine the effect that the strong absorption of hemoglobin has on HDP signal generation. Data corresponds to averages of six measurements. Error bars, mean ± SD. Source data are provided as a Source Data file.

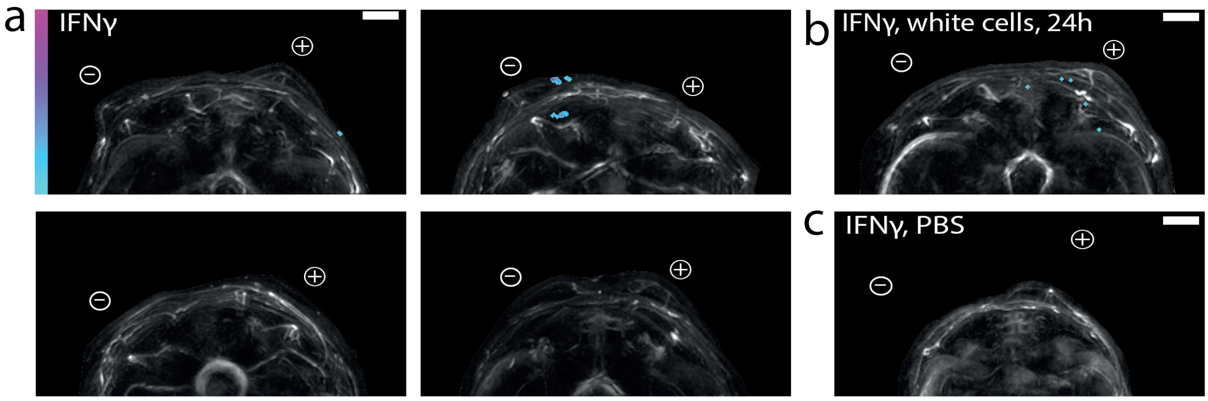

Suppl. Figure 12. Control animals analyzed in MSOT. (a) OA signal was determined in FoxN1 nude mice each carrying two dorsal matrigel implants, one with and one without IFNγ supplementation. (b) Non-labelled 'white' primary macrophages or (c) PBS were i.v. injected into animals and MSOT measurements were carried out 24 h later. Scale bar is 2 mm.

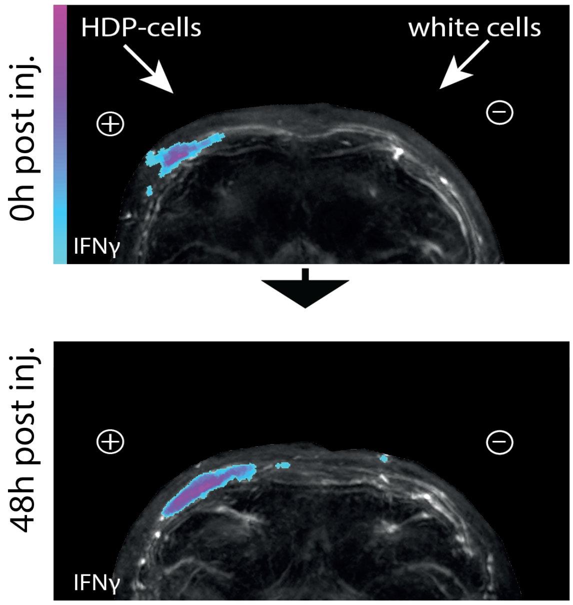

Suppl. Figure 13. OA signal stability of HDP-laden macrophages *in vivo*. Prolonged *in vivo* signal stability of the HDP-label in primary M1-activated macrophages is indicated 48h after dorsal implantation of $1x10^6$ labeled cells with IFN-γ-infused matrigel. Additionally, $1x10^6$ non-labeled ('white') macrophages were implanted on the same animal and served as a negative control.

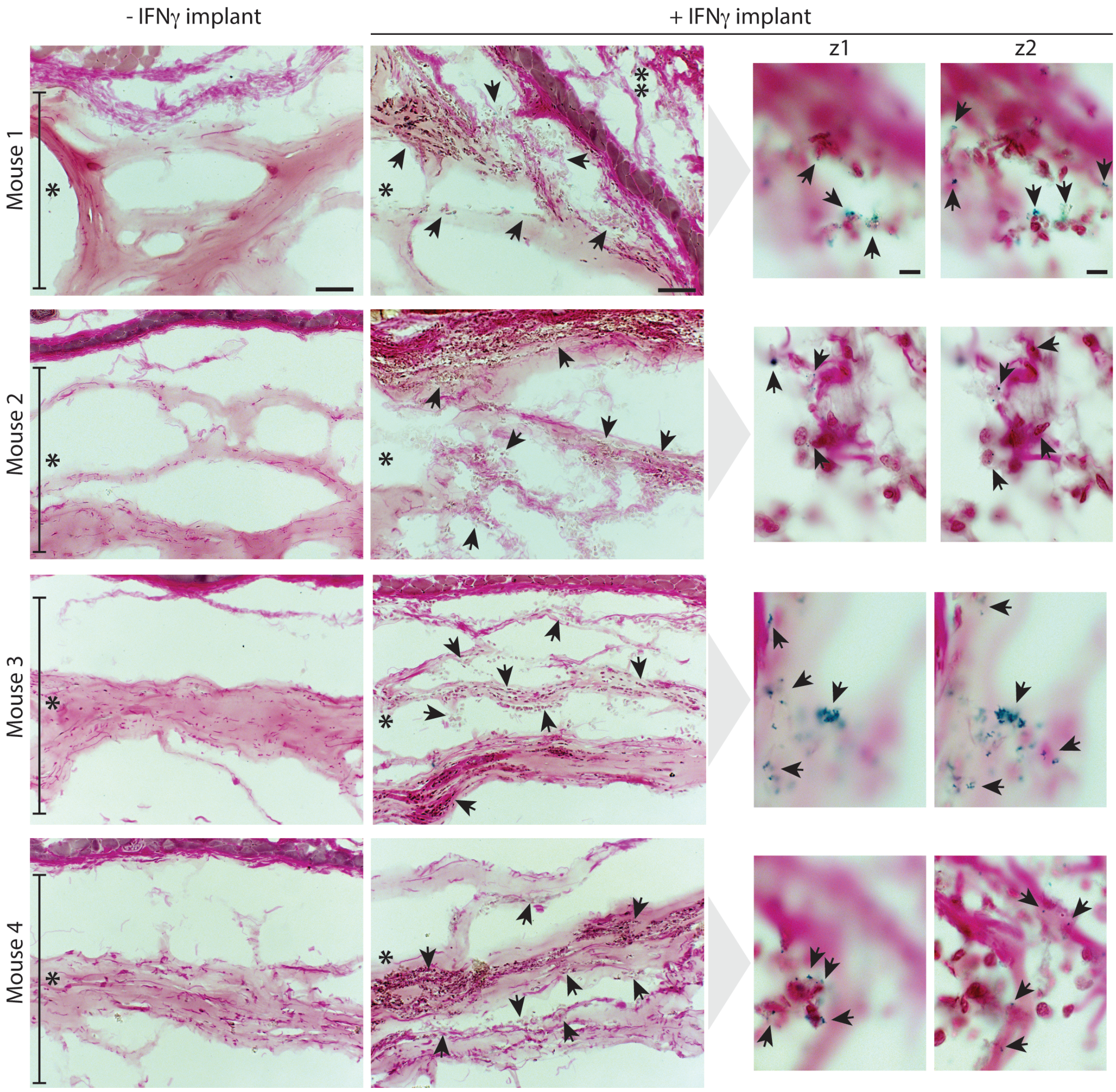

Suppl. Figure 14. Histological overview of the 4 recruitment animals. Histological tissue sections of the dorsal matrigel implants are presented 24h post cell injection and after Schmorl staining to highlight HDP in blue/green. Enrichment of the injected HDP-laden macrophages (arrows) is evident within and at the boundaries of the + IFNγ-supplemented matrigel (*) in all animals compared to their corresponding -IFNγ implants (*left*). Scale bars are 150 µm (20x objective). The small HDP-filled vesicles within cells are shown on the right in two different planes (z1 and z2). Z scans of 200nm step size were acquired with z1 and z2 chosen to be 2-5 µm apart. The HDP-filled cells are again indicated with arrows. It must be kept in mind that the macrophages have an average diameter of 20 µm and the tissue sections are of only 10 µm thickness. Consequently, only parts of a cell are represented at times. Scale bars are 10 µm (100x objective). Mouse numbers 1-4 directly correspond to f-i of Figure 3.

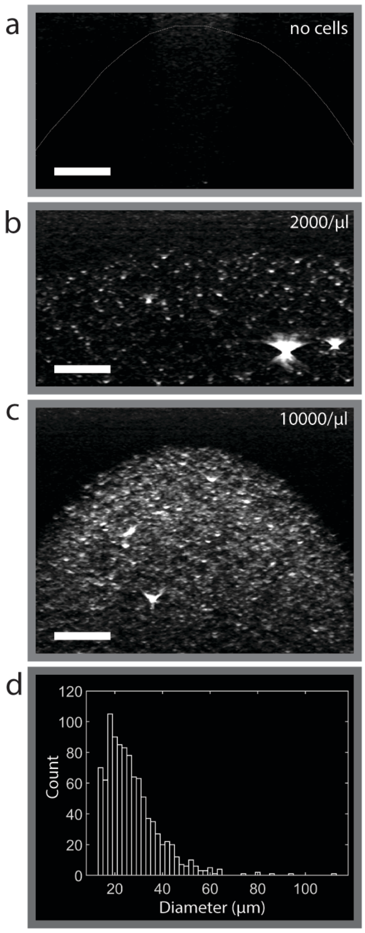

Suppl. Figure 15. HDP-mediated single cell visualization in blood-agar phantoms with Raster-scan optoacoustic Mesoscopy (RSOM). Depth-resolved maximum intensity projections (MIP) from OA volumetric data at 630 nm depicting absence of signal from the pure blood-agar phantom (a) and presence of signal for blood-agar phantom droplets with HDP-laden primary macrophages added at 2 000 (b) and 10 000 (c) cells/µl concentration. (d) The detected events from (c) follow a partial Gaussian size distribution with a mean diameter value of 27 µm and peak diameter of 20 µm, compliant with the average diameter of primary macrophages in solution. See Figure 5a for the volumetric scatter plot corresponding to (c and d).

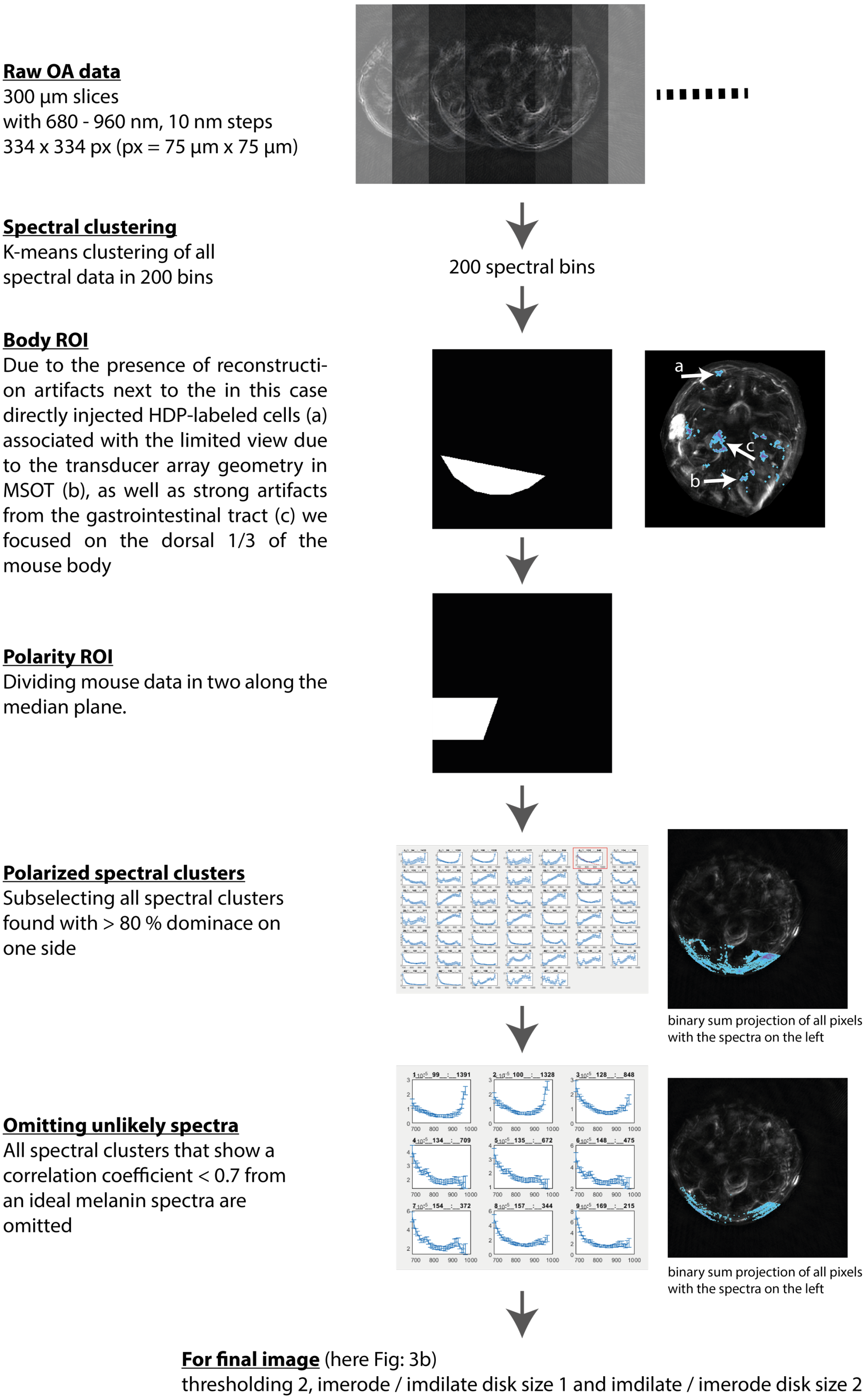

Suppl. Figure 16. Schematic of the analysis procedure for the MSOT data. Please note that the orientation of the mouse is different than in all other images in the manuscript; here the mouse belly is on the upper side of the image.

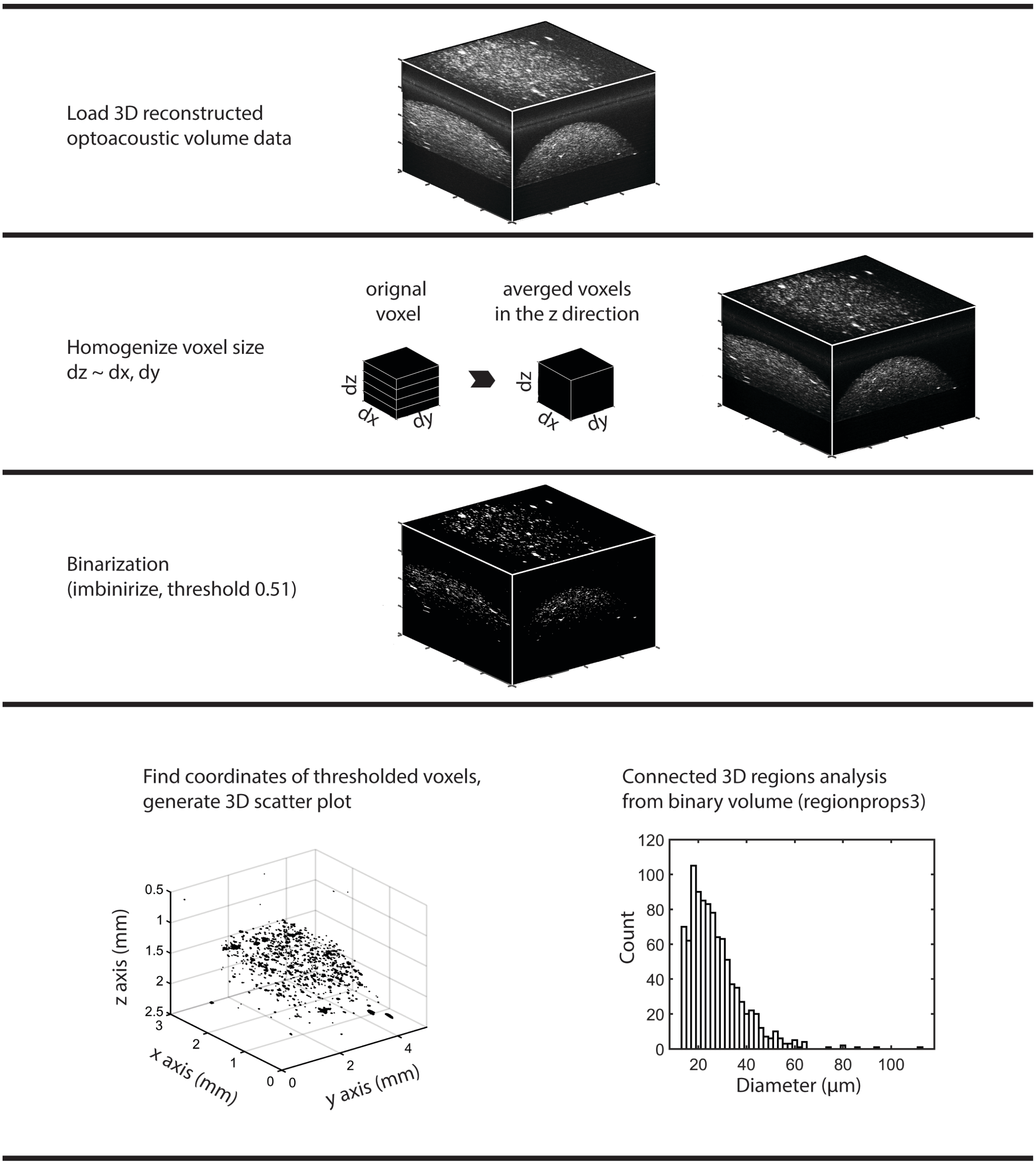

Suppl. Figure 17. Schematic of the analysis procedure for the RSOM *in vitro* blood-phantom data.

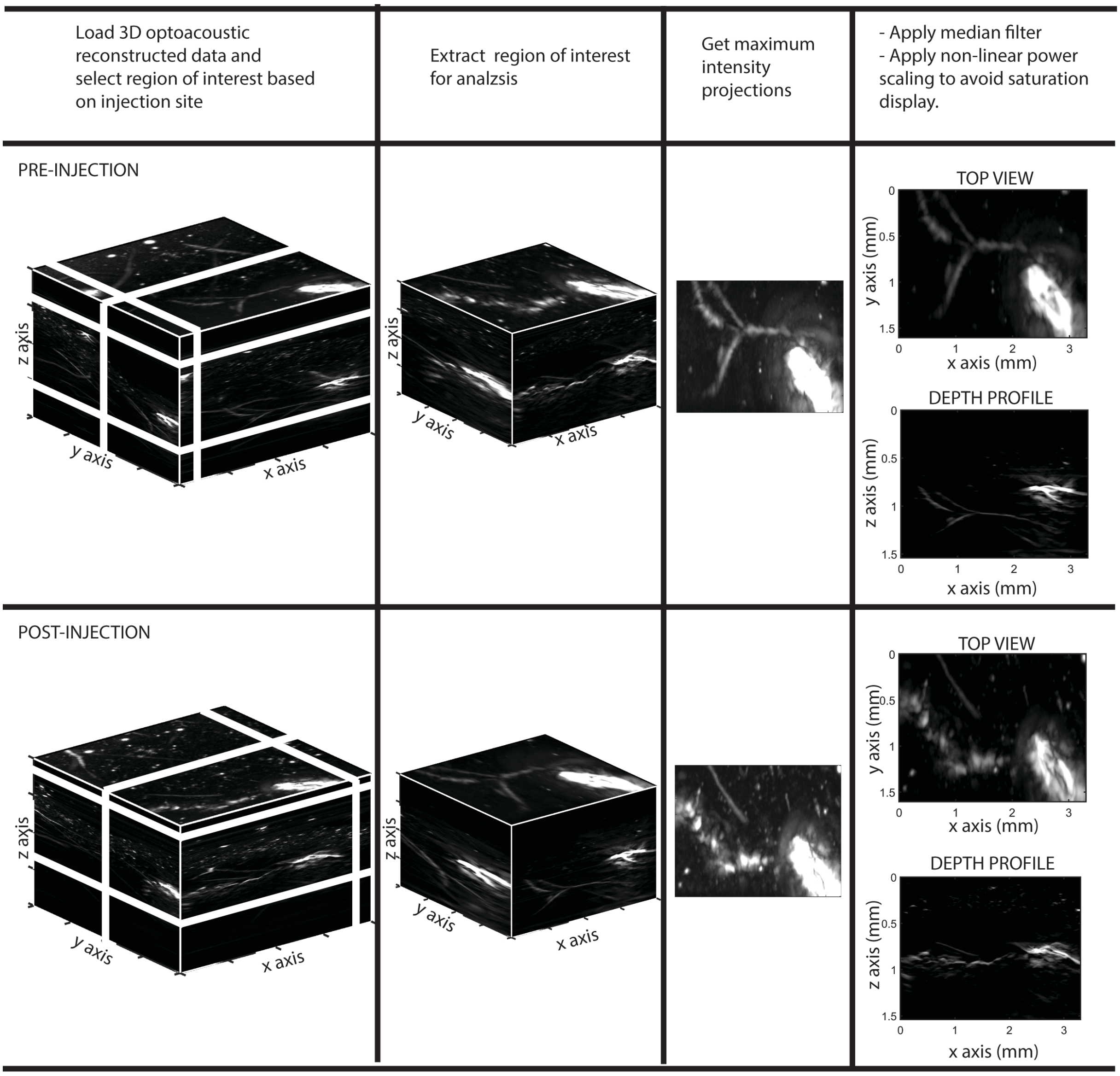

Suppl. Figure 18. Schematic of the analysis procedure for the RSOM *in vivo* data.

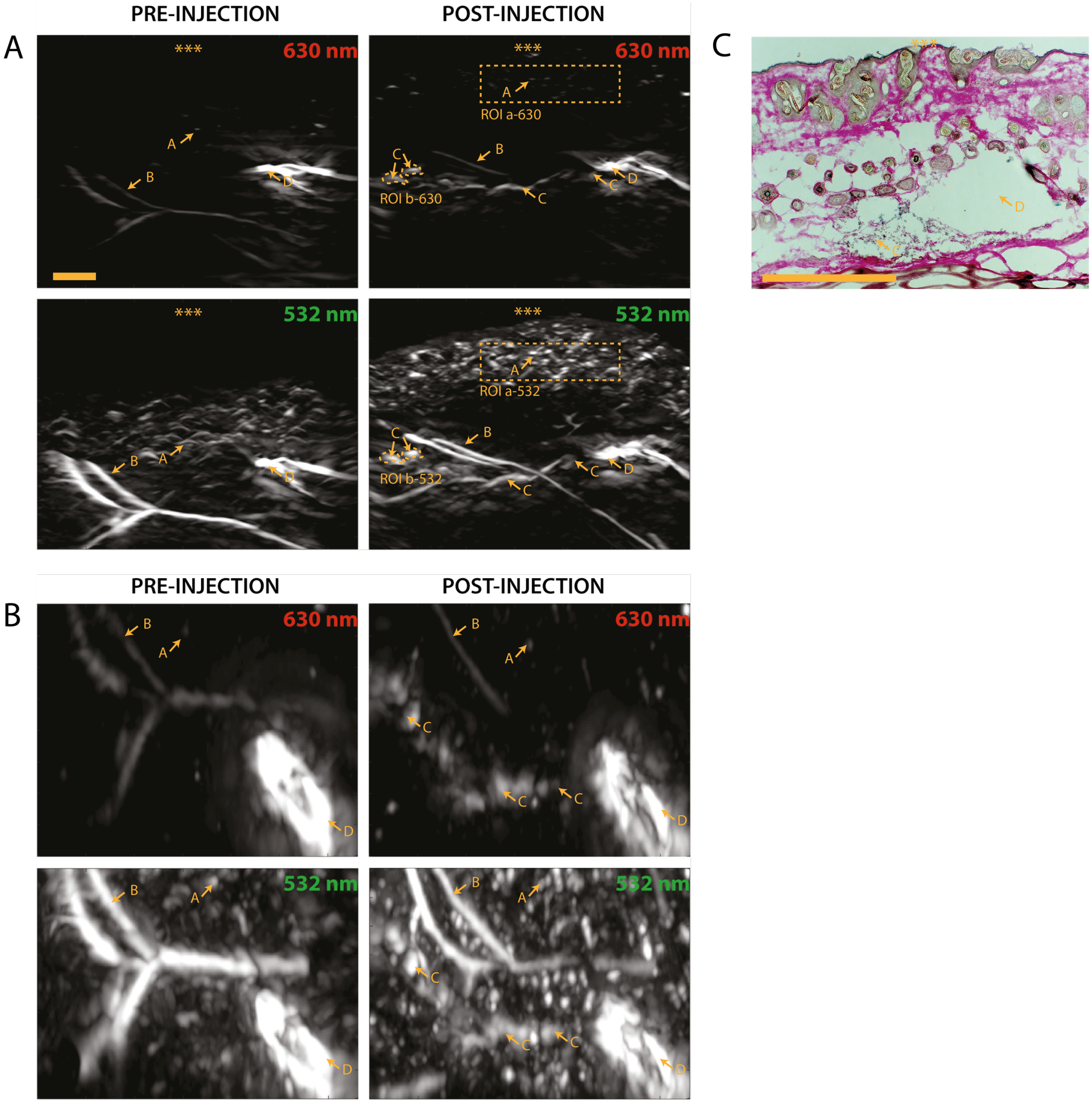

Suppl. Figure 19: 532 nm RSOM control wavelength images for the *in vivo* visualization of injected HDP-labeled Macrophages. 630 nm and 532 nm images shown as depth profile (**a**) and top view (**b**). The signal for blood hemoglobin and HDP-pigment at 532 nm and 630 nm allows us to confirm the presence of HDP-labeled macrophages (arrow C): For the capillary loops (hemoglobin) the intensity of the signal at 630 nm (ROI a-630, 7.7) is ~12% of the signal at 532 nm (ROI-a 532, 61,8). In contrast for the HDP-labeled macrophages the signal at 630 nm (ROI b-630, ~94.5) is ~53% of the signal at 532 nm (ROI b-532, ~173.5). The difference in the drop of signal intensity between 532 and 630 nm reflects the less pronounced descended of the melanin-like spectra in this wavelengths range compared to hemoglobin. The increase of signal for the capillary loops in the papillary dermis (arrow A) after injection is due to the inflation of the tissue as a result of injection. Conversely, for deeper vessels (arrow B) the light has to travel further now, thus they have a less strong signal in the post-injection data. (**c**) Histological tissue sections with Schmorl staining for the region near the needle tip (arrow D). In the histology it is apparent that, due to the layered structure of the skin, the HDP-labeled macrophages are restricted to only a small area (arrow C) while the solution can expand more freely leading to the expansion described above. Scale bars are 500 µm in all panels in x,y and z.
