# Supplementary material for: Homogentisic acid-derived pigment as a biocompatible label for optoacoustic imaging of macrophages
Source: Nat Commun. 2019 Nov 7;10:5056. doi: 10.1038/s41467-019-13041-4 (PMC6838096; doi:10.1038/s41467-019-13041-4)
Supplement: Supplementary file 3 — Reporting Summary [file 41467_2019_13041_MOESM3_ESM.pdf]

nature research

Corresponding author(s): STIEL (NCOMMS-18-29447A)

Last updated by author(s): 04, August, 2019

Reporting Summary

Nature Research wishes to improve the reproducibility of the work that we publish. This form provides structure for consistency and transparency in reporting. For further information on Nature Research policies, see Authors & Referees and the Editorial Policy Checklist.

Statistics

For all statistical analyses, confirm that the following items are present in the figure legend, table legend, main text, or Methods section.

n/a	Confirmed	
☐	☒	The exact sample size (n) for each experimental group/condition, given as a discrete number and unit of measurement
☐	☒	A statement on whether measurements were taken from distinct samples or whether the same sample was measured repeatedly
☐	☒	The statistical test(s) used AND whether they are one- or two-sided<br>*Only common tests should be described solely by name; describe more complex techniques in the Methods section.*
☐	☒	A description of all covariates tested
☒	☐	A description of any assumptions or corrections, such as tests of normality and adjustment for multiple comparisons
☒	☐	A full description of the statistical parameters including central tendency (e.g. means) or other basic estimates (e.g. regression coefficient) AND variation (e.g. standard deviation) or associated estimates of uncertainty (e.g. confidence intervals)
☒	☐	For null hypothesis testing, the test statistic (e.g. F, t, r) with confidence intervals, effect sizes, degrees of freedom and P value noted<br>*Give P values as exact values whenever suitable.*
☒	☐	For Bayesian analysis, information on the choice of priors and Markov chain Monte Carlo settings
☒	☐	For hierarchical and complex designs, identification of the appropriate level for tests and full reporting of outcomes
☒	☐	Estimates of effect sizes (e.g. Cohen's d, Pearson's r), indicating how they were calculated

Our web collection on statistics for biologists contains articles on many of the points above.

Software and code

Policy information about availability of computer code

Data collection	All MSOT data was collected using ViewMSOT 3.8; RSOM and general reconstruction and unmixing was carried out by custom code as described and refenced in the MS. Light microcopy images were acquired and processed using the corresponding Zeiss ZEN (blue edition) software. Multiplex data was collected and analyzed on a MAGPIX® system from Merck.
Data analysis	Image processing of MSOT and RSOM was carried out with Matlab R2017 and ImageJ 1.52e. Light microcopy images were processed using the corresponding Zeiss ZEN (blue edition) software. FACS data was analyzed using FlowJo10. Multiplex data was analyzed on a MAGPIX® system from Merck. All other data analyses was done either with excel 2013 or Graphpad Prism7.

For manuscripts utilizing custom algorithms or software that are central to the research but not yet described in published literature, software must be made available to editors/reviewers. We strongly encourage code deposition in a community repository (e.g. GitHub). See the Nature Research guidelines for submitting code & software for further information.

Data

Policy information about availability of data

All manuscripts must include a data availability statement. This statement should provide the following information, where applicable:

- Accession codes, unique identifiers, or web links for publicly available datasets
- A list of figures that have associated raw data
- A description of any restrictions on data availability

There is no restriction on data availability. All figures have associated raw data.

Field-specific reporting

Please select the one below that is the best fit for your research. If you are not sure, read the appropriate sections before making your selection.

☒ Life sciences ☐ Behavioural & social sciences ☐ Ecological, evolutionary & environmental sciences

For a reference copy of the document with all sections, see nature.com/documents/nr-reporting-summary-flat.pdf

Life sciences study design

All studies must disclose on these points even when the disclosure is negative.

Sample size	All in vitro measurements have been carried out as triplicates unless stated otherwise.<br>Further, for each MSOT cell sample, 5-7 different positions within the sample were acquired. For MSOT animal studies: Functional Recruitment to IFNgamma positive sites with n=4. In addition, internal negative controls (IFN negative implant) for each of the 4 animals. For internal detectability of HDP-cells locally injected n=5. Different negative controls with non-labeled cells, PBS or implants without cells n=6. RSOM data set conducted with n=3. In vivo RSOM shown as n=1, with data of the identical region shown before and after cell injection. Analysis of animal serum of n=3 mice injected with HDP-labeled macrophages.
Data exclusions	No data was excluded.
Replication	Please see 'Sample Size' for replications
Randomization	For the the in vivo experiments allocation of mice to the different groups / experiments was fully random.
Blinding	Blinding was conducted for the MSOT studies for unmixing and identifying OA signals in animals by not disclosing the dataset/experiment or group the data belonged to , to the person performing the analysis.

Reporting for specific materials, systems and methods

We require information from authors about some types of materials, experimental systems and methods used in many studies. Here, indicate whether each material, system or method listed is relevant to your study. If you are not sure if a list item applies to your research, read the appropriate section before selecting a response.

Materials & experimental systems

n/a	Involved in the study	
☐	☒	Antibodies
☐	☒	Eukaryotic cell lines
☐	☐	Palaeontology
☐	☒	Animals and other organisms
☐	☐	Human research participants
☐	☐	Clinical data

Methods

n/a	Involved in the study	
☒	☐	ChIP-seq
☐	☒	Flow cytometry
☒	☐	MRI-based neuroimaging

Antibodies

Antibodies used	F4/80-APC (Kat# 17-4801, Klon BM8, Lot. 4271644, Affimetrix); CD38-FITC Source = Glasmacher HMGU), CD11b-FITC (Kat# 11-0112, Klon M1/70, Lot. E00147-1633, Affimetrix), Lamp1 (Kat# 24170, Rb polyclonal, Abcam); Procarta Plex Mix&Match Mouse (Invitrogen), Ref: PPX-24-MXFVKT3, Lot: 166170000; SAA Elisa R&D-Systems, Ref: DY2948-05
Validation	See manufacturers website

Eukaryotic cell lines

Policy information about cell lines

Cell line source(s)	Ana-1 macrophage cell line (Source is Heiko Adler, HMGU). Primary bone marrow derived macrophages (BMDM) have been obtained in our cell culture from FoxN1 nude female mice aged 8-10 wks (Charles River Labs, Boston, USA)
Authentication	Primary BMDMs and activation state have been confirmed with above listed ABs.
Mycoplasma contamination	not tested for BMDMs due to their application within 12 days post isolation of bone marrow cells.

Commonly misidentified lines (See ICLAC register) | not applicable

Palaeontology

Specimen provenance | *Provide provenance information for specimens and describe permits that were obtained for the work (including the name of the issuing authority, the date of issue, and any identifying information).*

Specimen deposition | *Indicate where the specimens have been deposited to permit free access by other researchers.*

Dating methods | *If new dates are provided, describe how they were obtained (e.g. collection, storage, sample pretreatment and measurement), where they were obtained (i.e. lab name), the calibration program and the protocol for quality assurance OR state that no new dates are provided.*

☐ Tick this box to confirm that the raw and calibrated dates are available in the paper or in Supplementary Information.

Animals and other organisms

Policy information about studies involving animals; ARRIVE guidelines recommended for reporting animal research

Laboratory animals | Fox N1 nude female mice (Charles River Labs, Boston, USA), age 8-10 wks.

Wild animals | not applicable

Field-collected samples | not applicable

Ethics oversight | All animal experiments were approved by the government of Upper Bavaria, Germany, and were carried out in accordance with official guidelines.

Note that full information on the approval of the study protocol must also be provided in the manuscript.

Human research participants

Policy information about studies involving human research participants

Population characteristics | *Describe the covariate-relevant population characteristics of the human research participants (e.g. age, gender, genotypic information, past and current diagnosis and treatment categories). If you filled out the behavioural & social sciences study design questions and have nothing to add here, write "See above."*

Recruitment | *Describe how participants were recruited. Outline any potential self-selection bias or other biases that may be present and how these are likely to impact results.*

Ethics oversight | *Identify the organization(s) that approved the study protocol.*

Note that full information on the approval of the study protocol must also be provided in the manuscript.

Clinical data

Policy information about clinical studies

All manuscripts should comply with the ICMJE guidelines for publication of clinical research and a completed CONSORT checklist must be included with all submissions.

Clinical trial registration | *Provide the trial registration number from ClinicalTrials.gov or an equivalent agency.*

Study protocol | *Note where the full trial protocol can be accessed OR if not available, explain why.*

Data collection | *Describe the settings and locales of data collection, noting the time periods of recruitment and data collection.*

Outcomes | *Describe how you pre-defined primary and secondary outcome measures and how you assessed these measures.*

Flow Cytometry

Plots

Confirm that:

☒ The axis labels state the marker and fluorochrome used (e.g. CD4-FITC).

☒ The axis scales are clearly visible. Include numbers along axes only for bottom left plot of group (a 'group' is an analysis of identical markers).

☒ All plots are contour plots with outliers or pseudocolor plots.

☒ A numerical value for number of cells or percentage (with statistics) is provided.

Methodology

Sample preparation	Primary bone-marrow derived macrophages were utilized and their preparation and AB staining for FACS is described in the manuscript.
Instrument	BD LSR Fortessa
Software	FlowJO 10 software
Cell population abundance	Cytometry was used to confirm the purity of BMDMs. The cells showed a purity of >99% as determined by CD11b and F4/80.
Gating strategy	we compare +HDP to -HDP cells (=wildtype). Further we compare unstained (no AB treatm.) for both to the different ABs listed above. We defined the unstained populations and compare them to the AB-stained populations. The gates and % are marked in the figure. No cell sorting was conducted.

☒ Tick this box to confirm that a figure exemplifying the gating strategy is provided in the Supplementary Information.
