# Supplementary material for: Homogentisic acid-derived pigment as a biocompatible label for optoacoustic imaging of macrophages
Source: Nat Commun. 2019 Nov 7;10:5056. doi: 10.1038/s41467-019-13041-4 (PMC6838096; doi:10.1038/s41467-019-13041-4)

MSOT raw data:

Shown are individual slices always first the unmixed binary slices and then the anatomy slice. No thresholding, or postprocessing on this data. All processing as described in material & methods and Suppl. Figure S16. Raw data matrices (20 GB) and Matlab code can be obtained from the authors upon request. Each page corresponds to one dataset shown in the manuscript:

Page:

1. Fig. 3a
2. Fig. 3e
3. Fig. 3d
4. Not used in manuscript, additional muscle injection
5. Fig. 3b
6. Fig. 3c
7. Fig. 3g
8. Fig. 3f
9. Fig. 3i
10. Fig. 3h
11. Fig. S13 top
12. Fig. S13 bottom
13. Fig. S12a bottom right
14. Fig. S12a bottom left
15. Fig. S12a top left
16. Fig. S12a top right
17. Fig. S12c
18. Fig. S12b & Fig. 3j

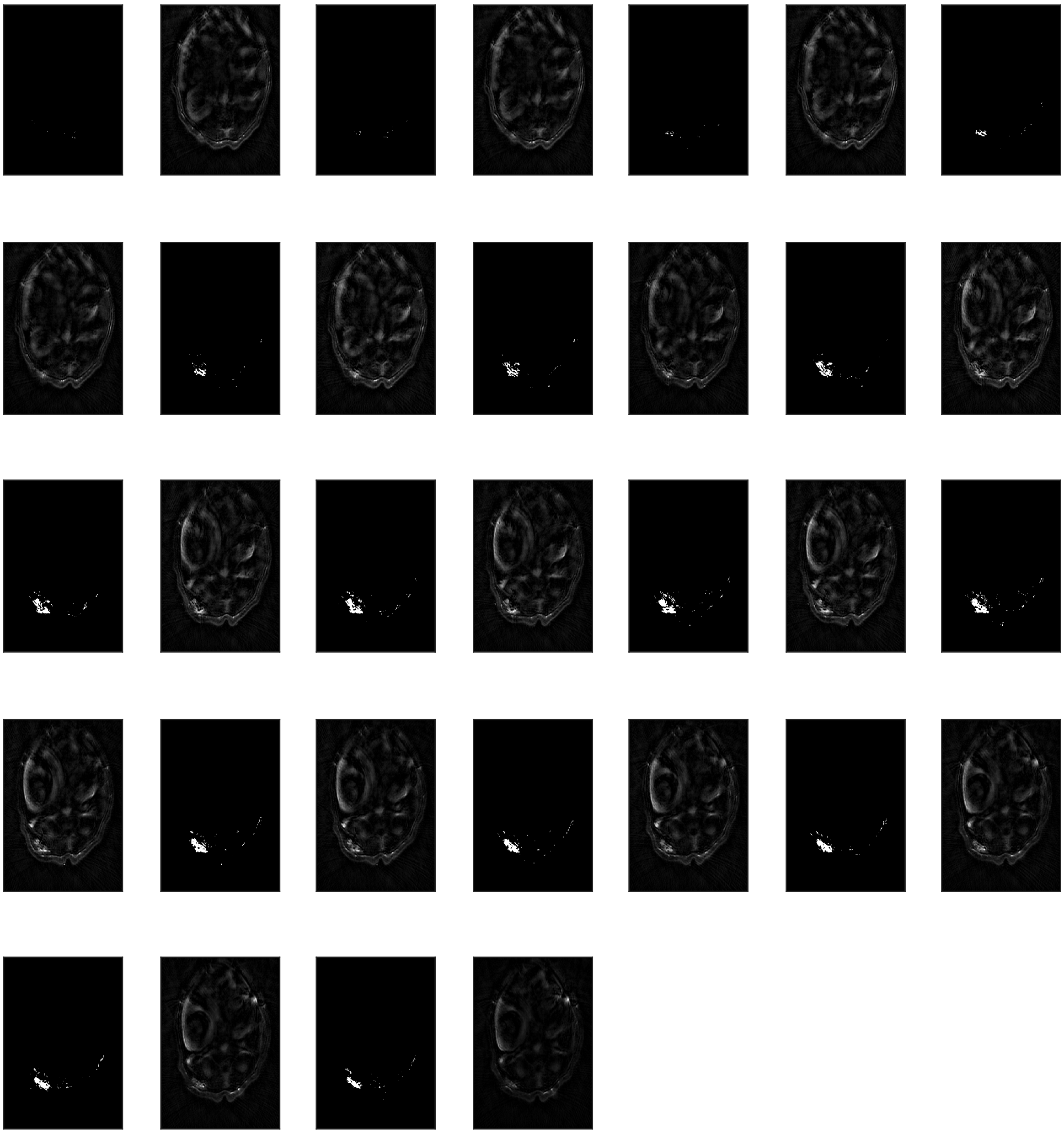

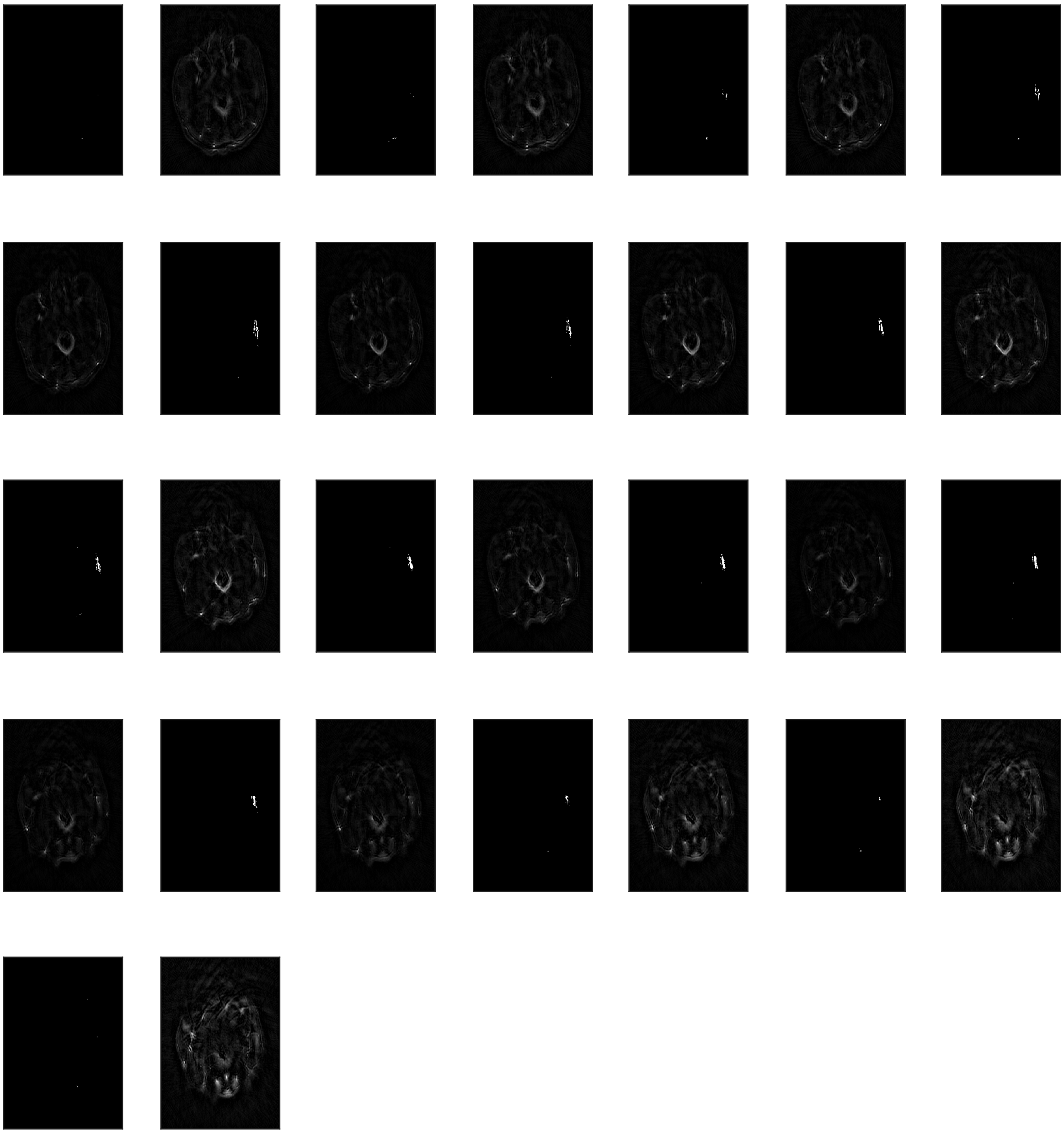

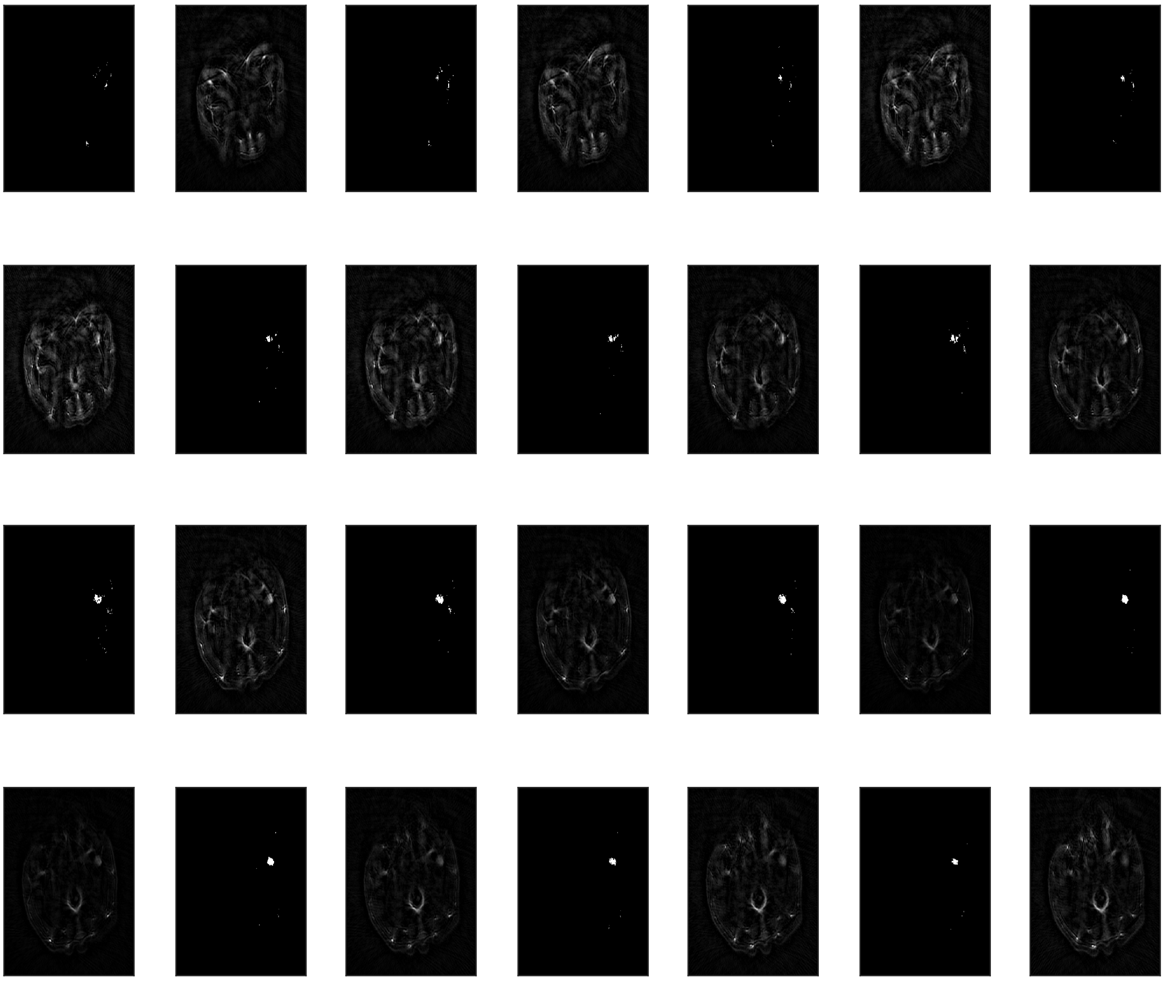

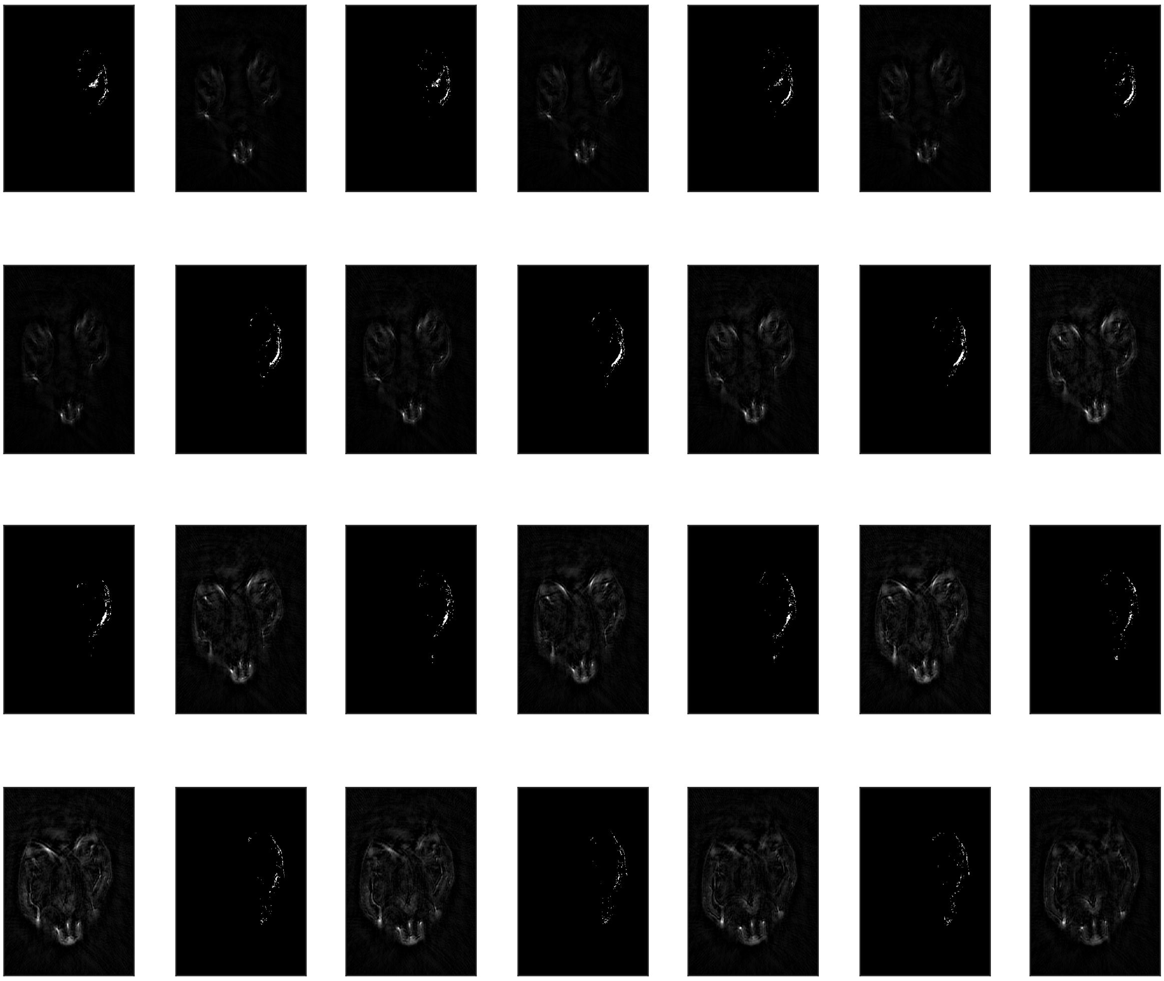

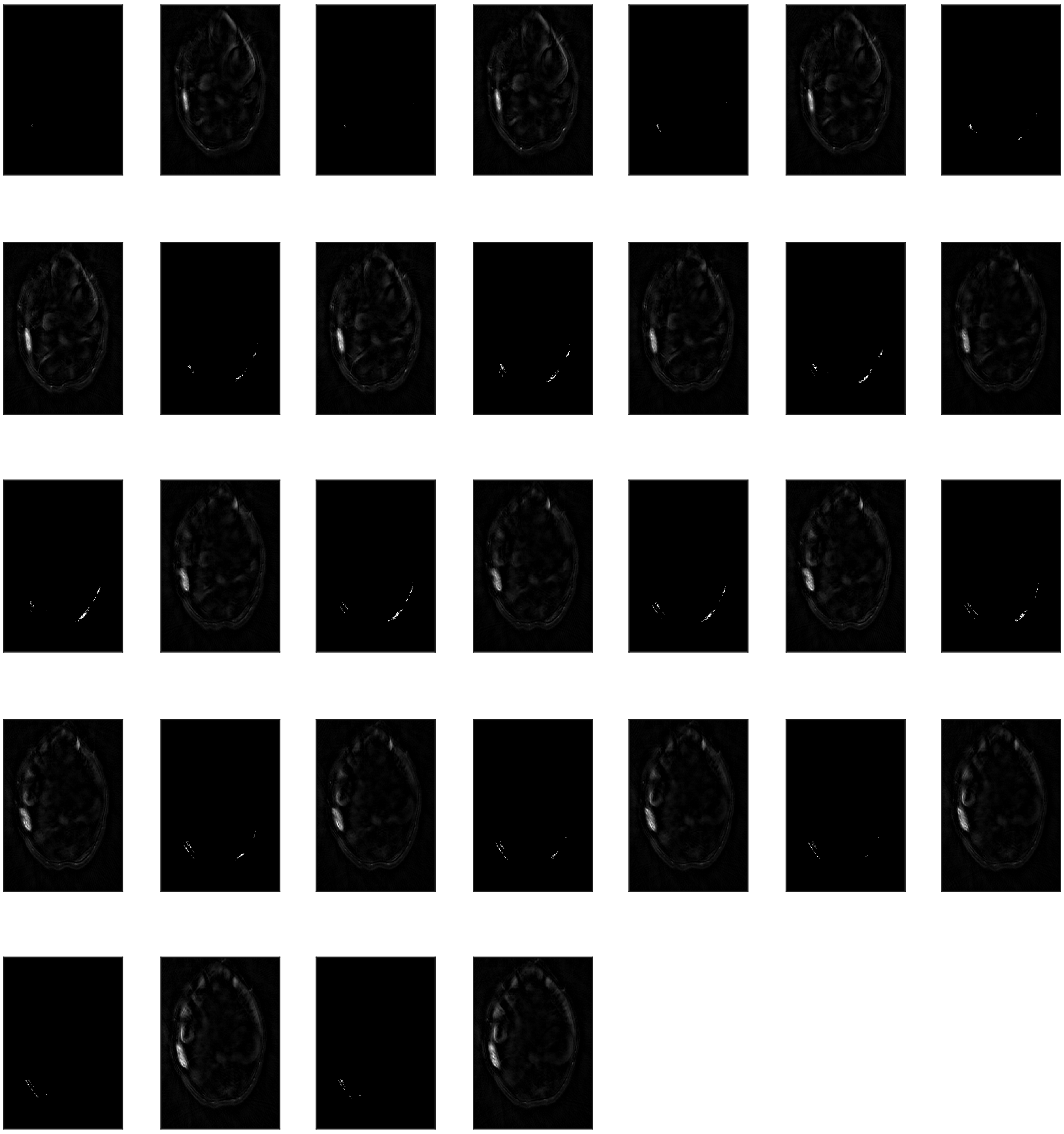

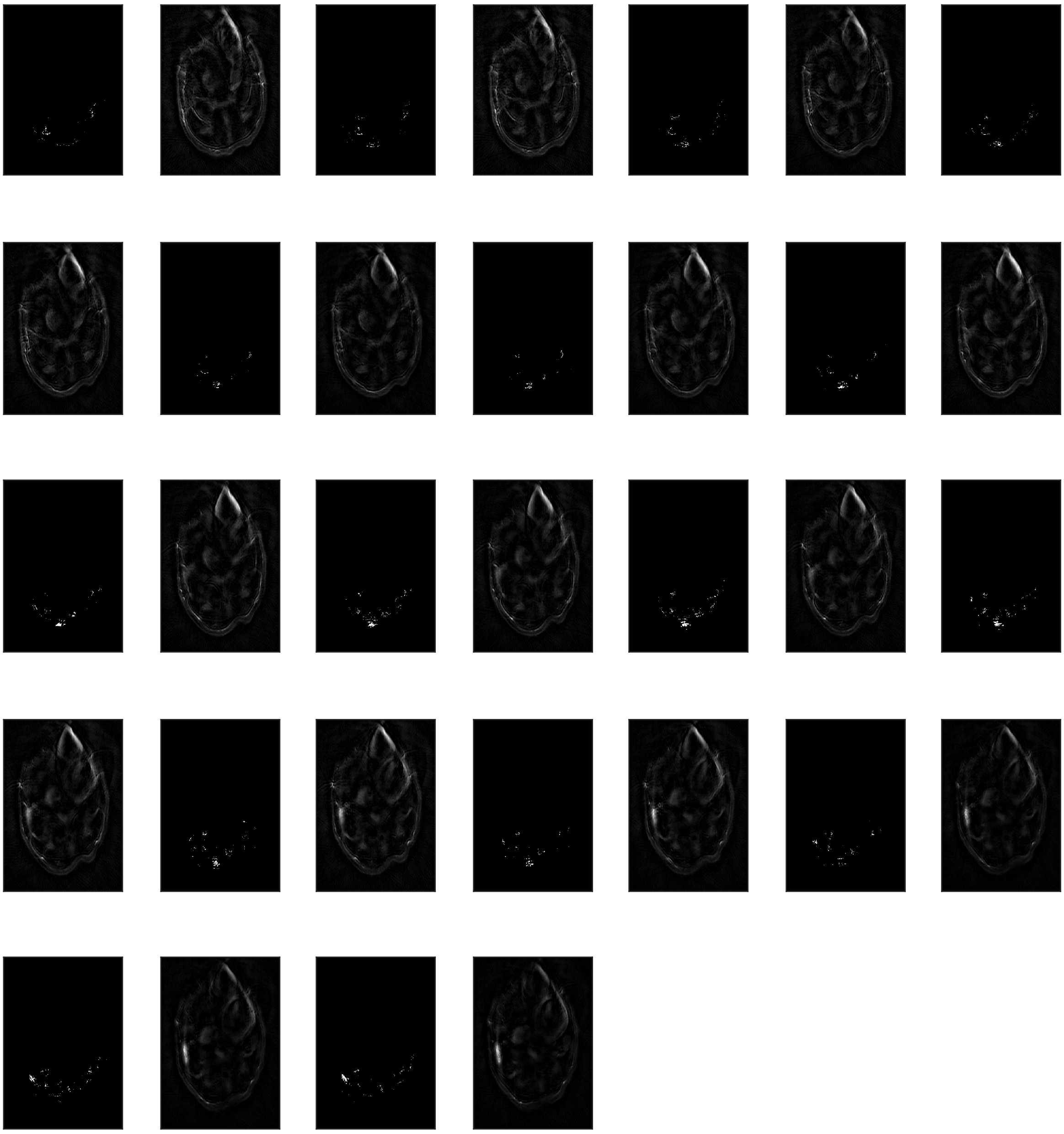

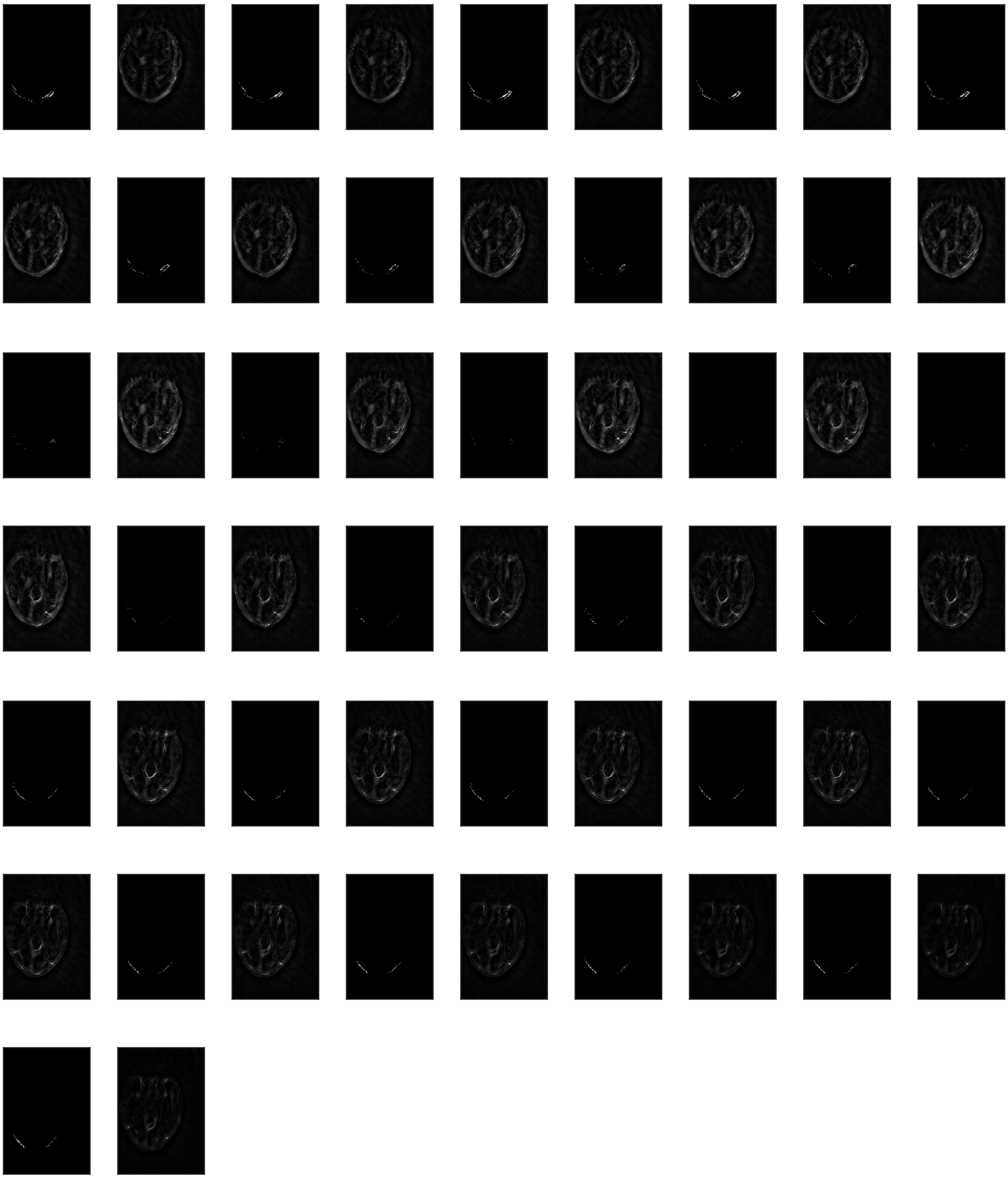

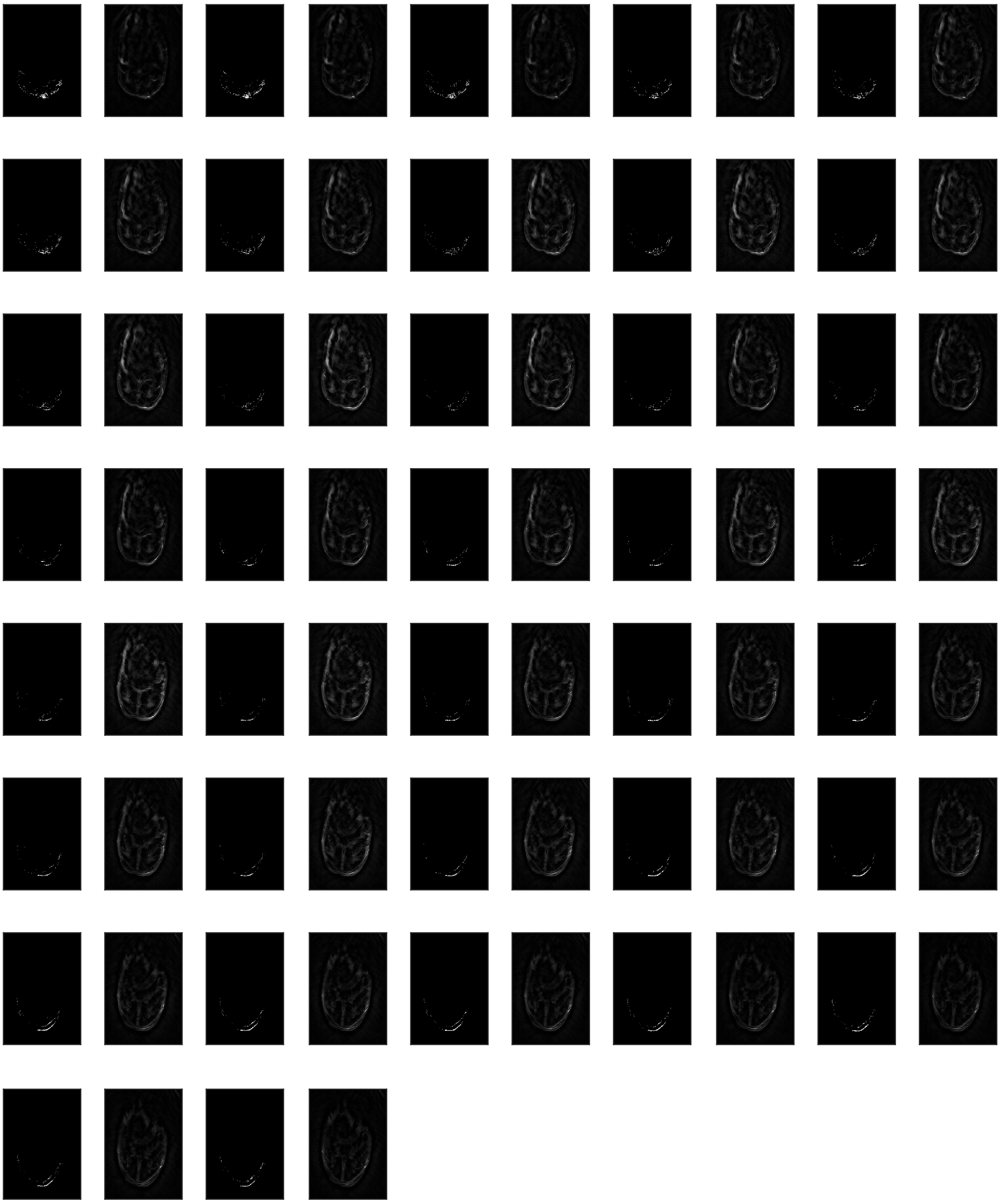

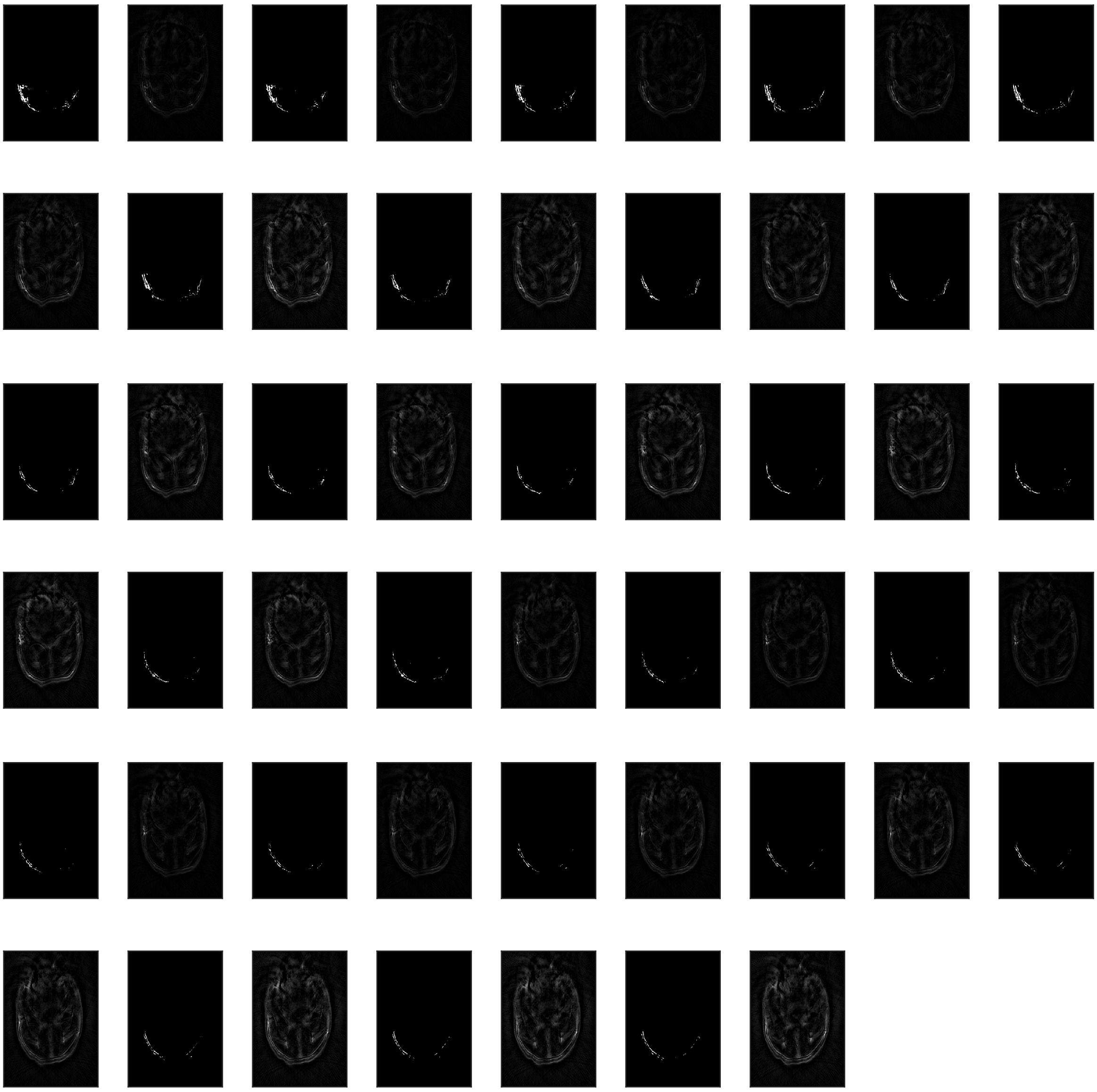

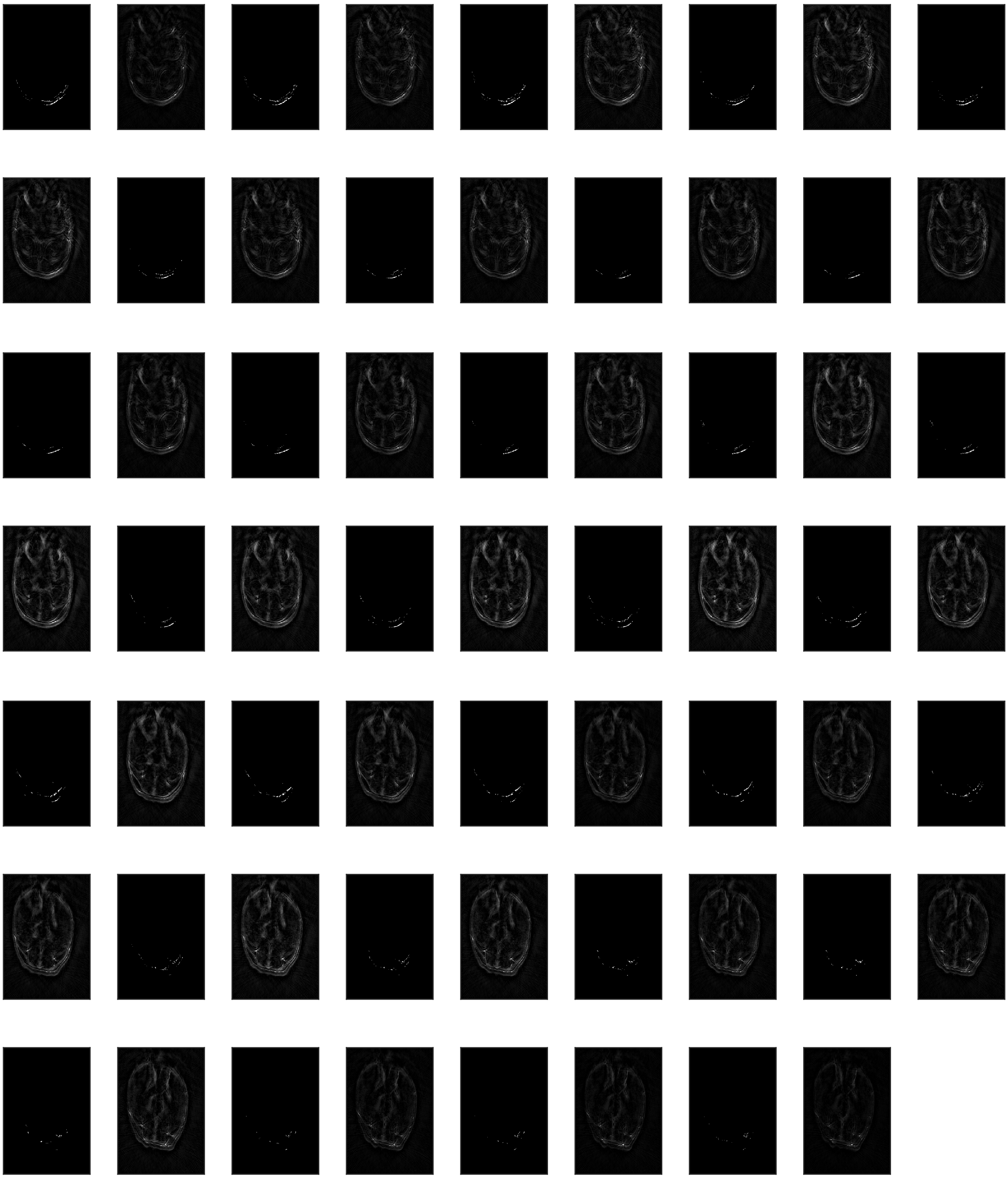

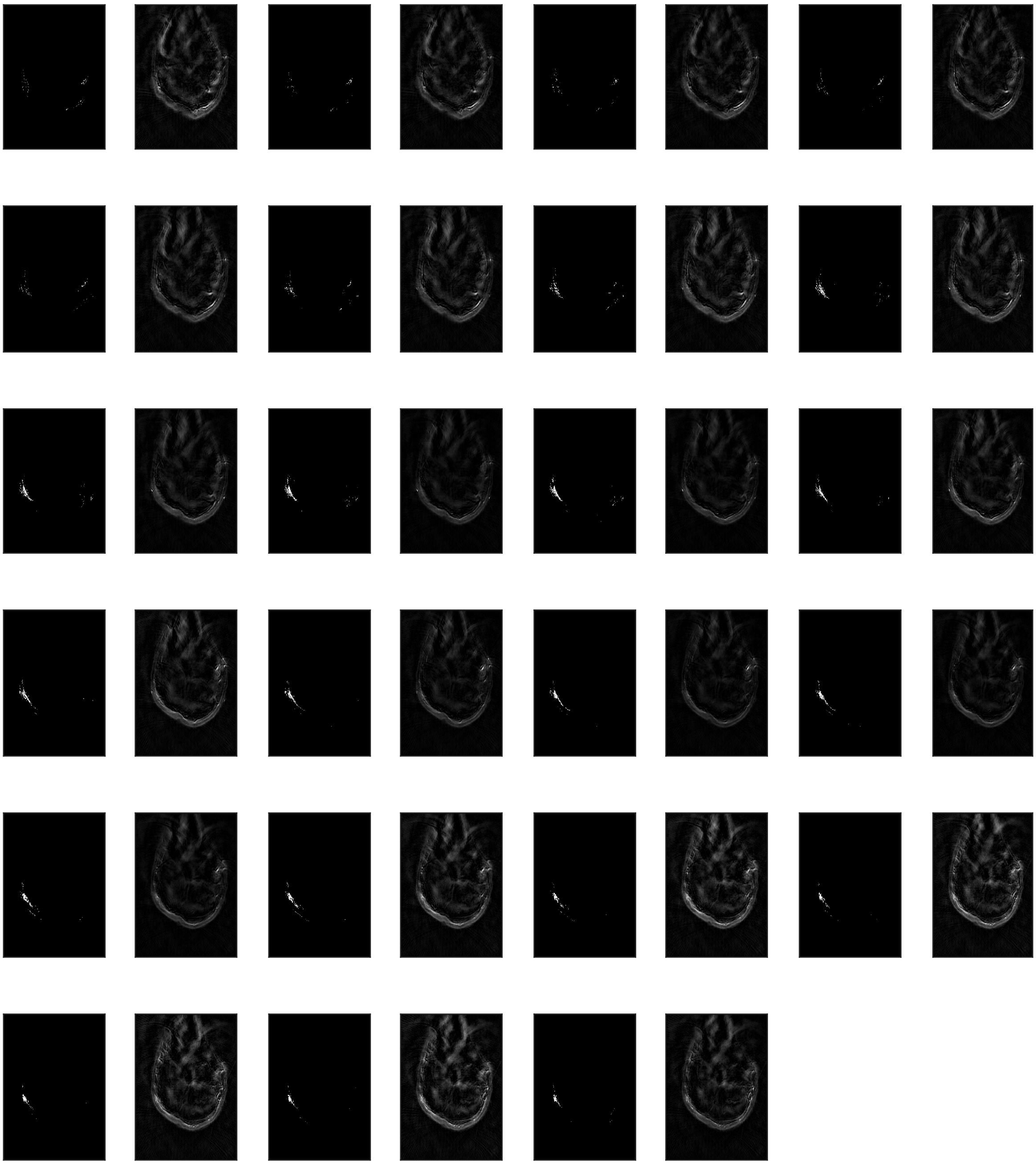

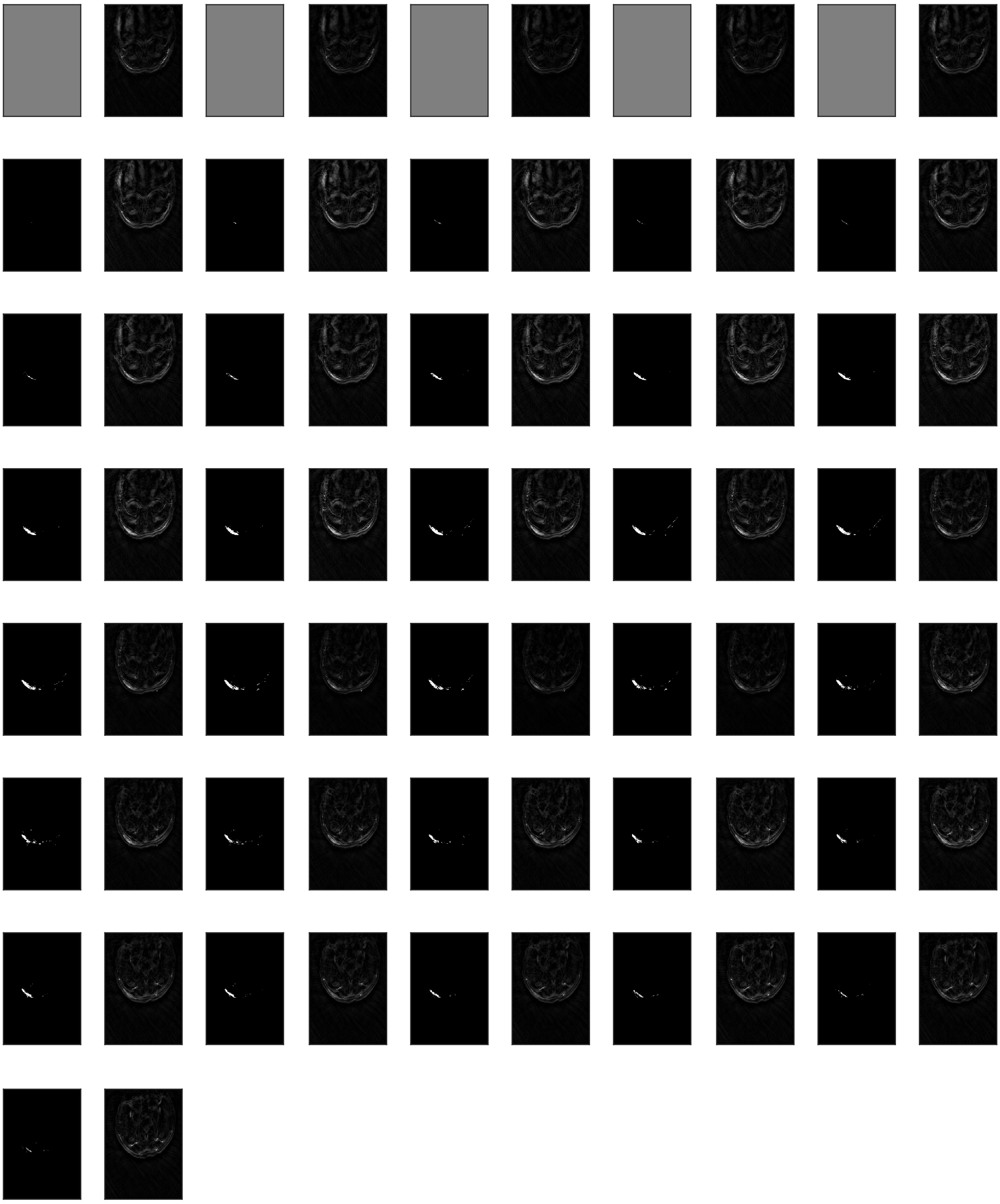

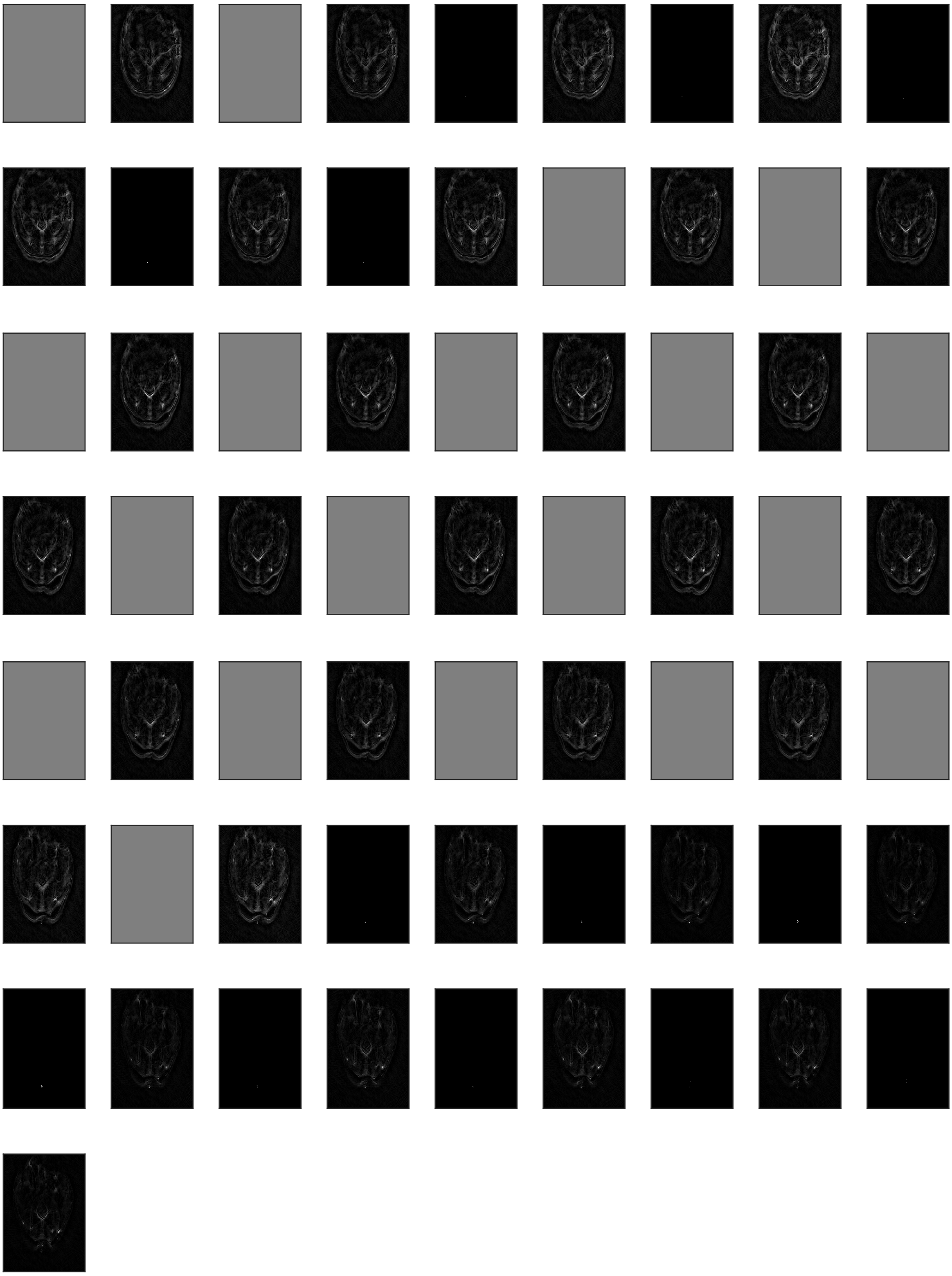

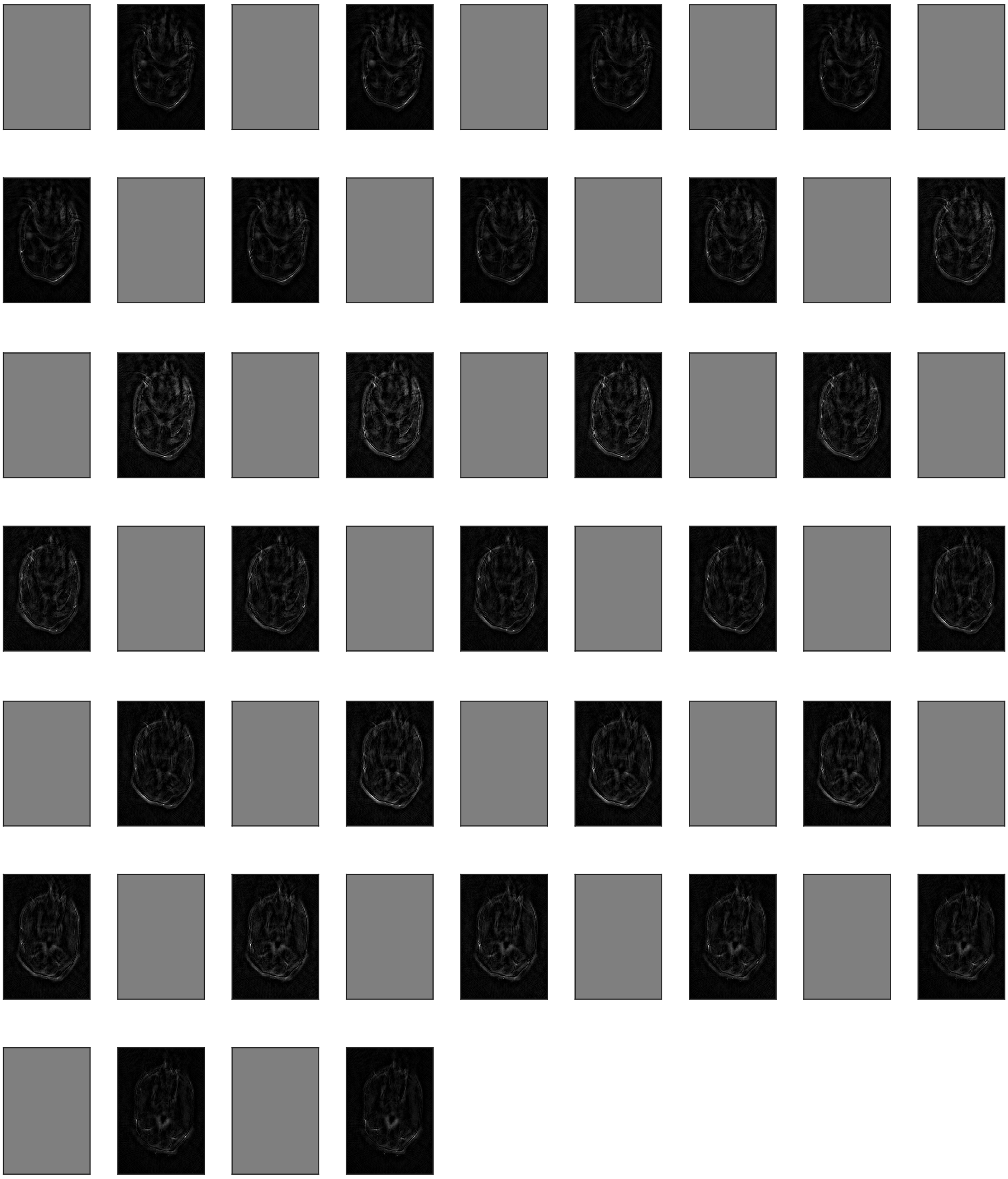

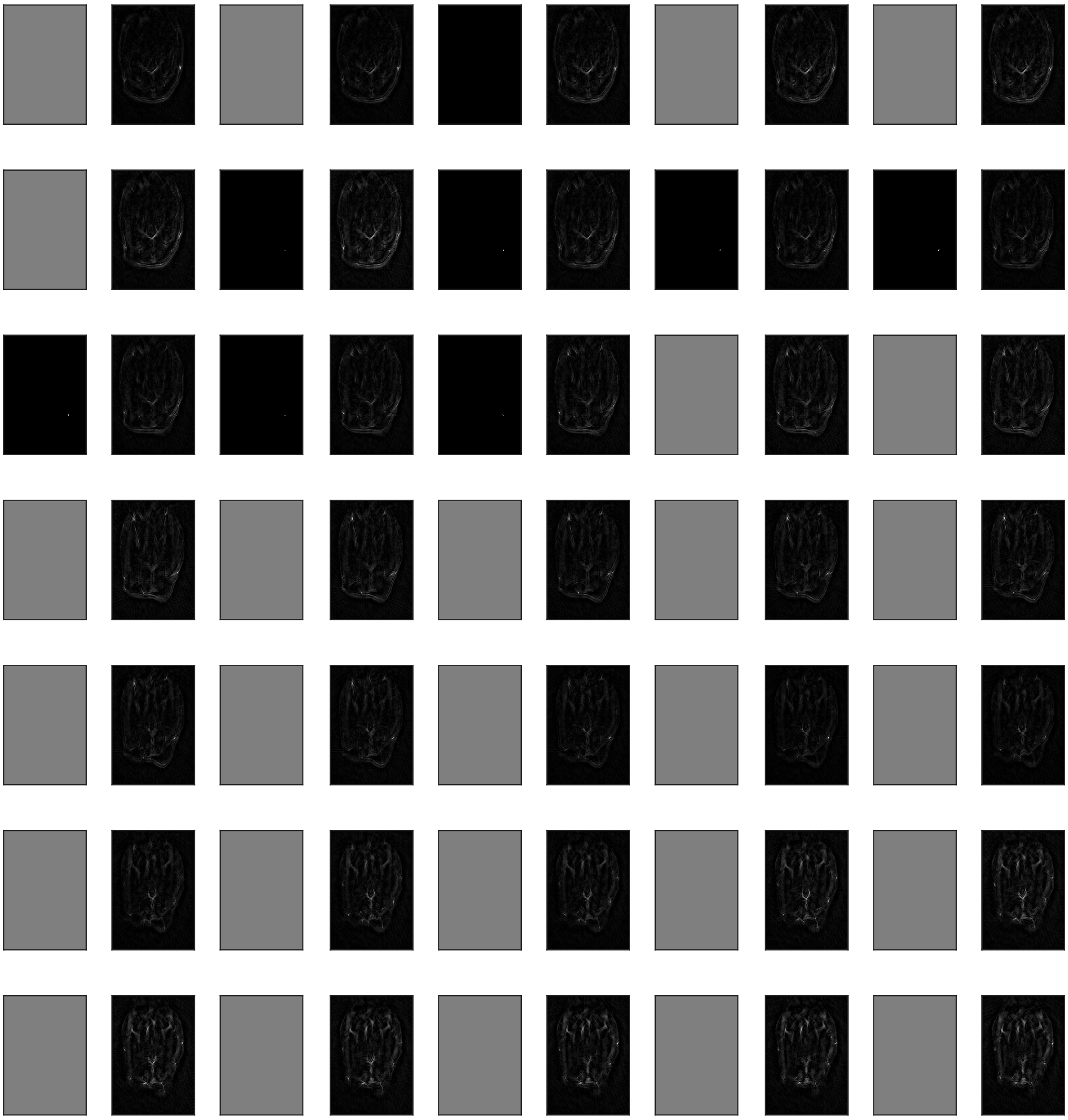

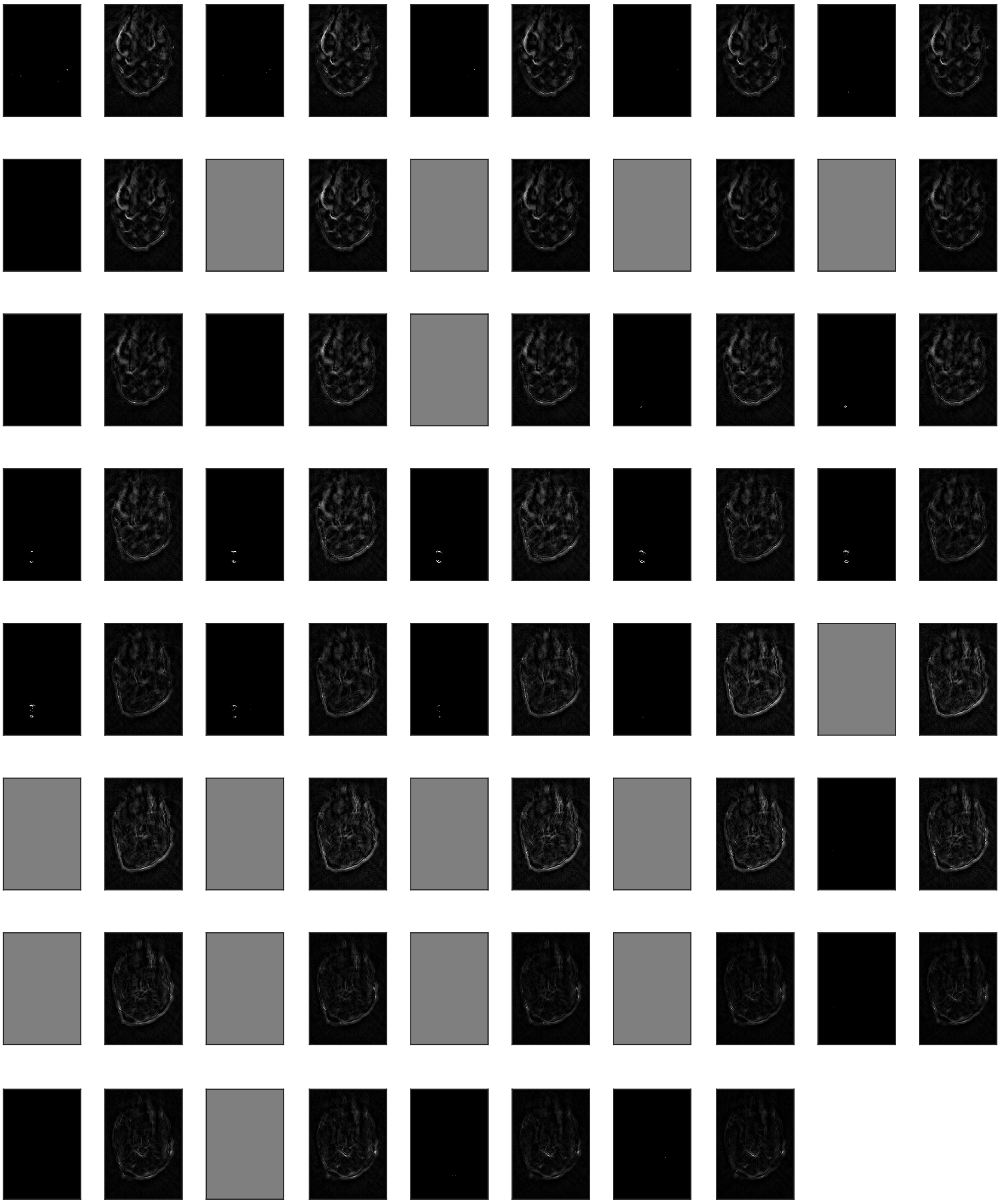

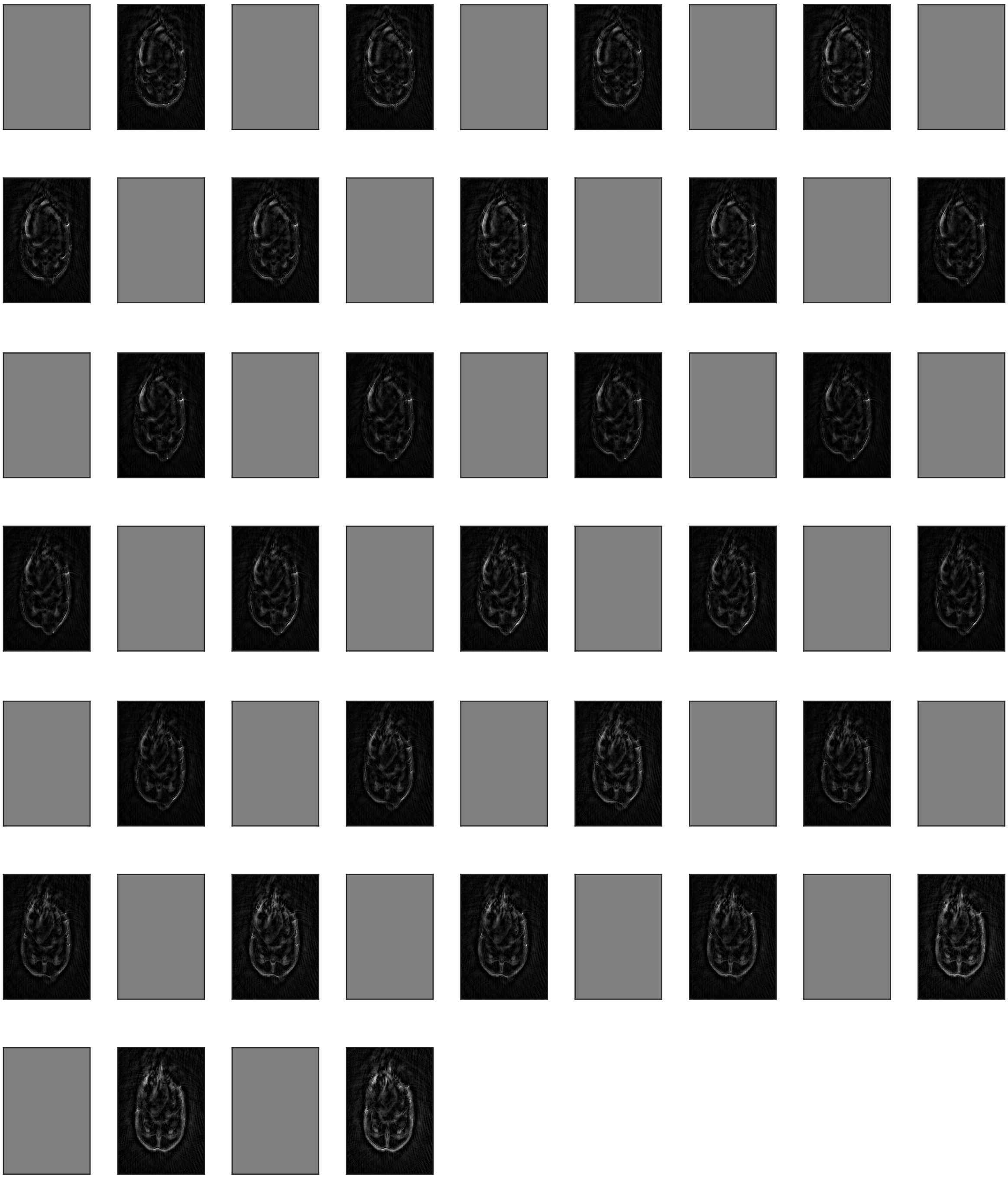

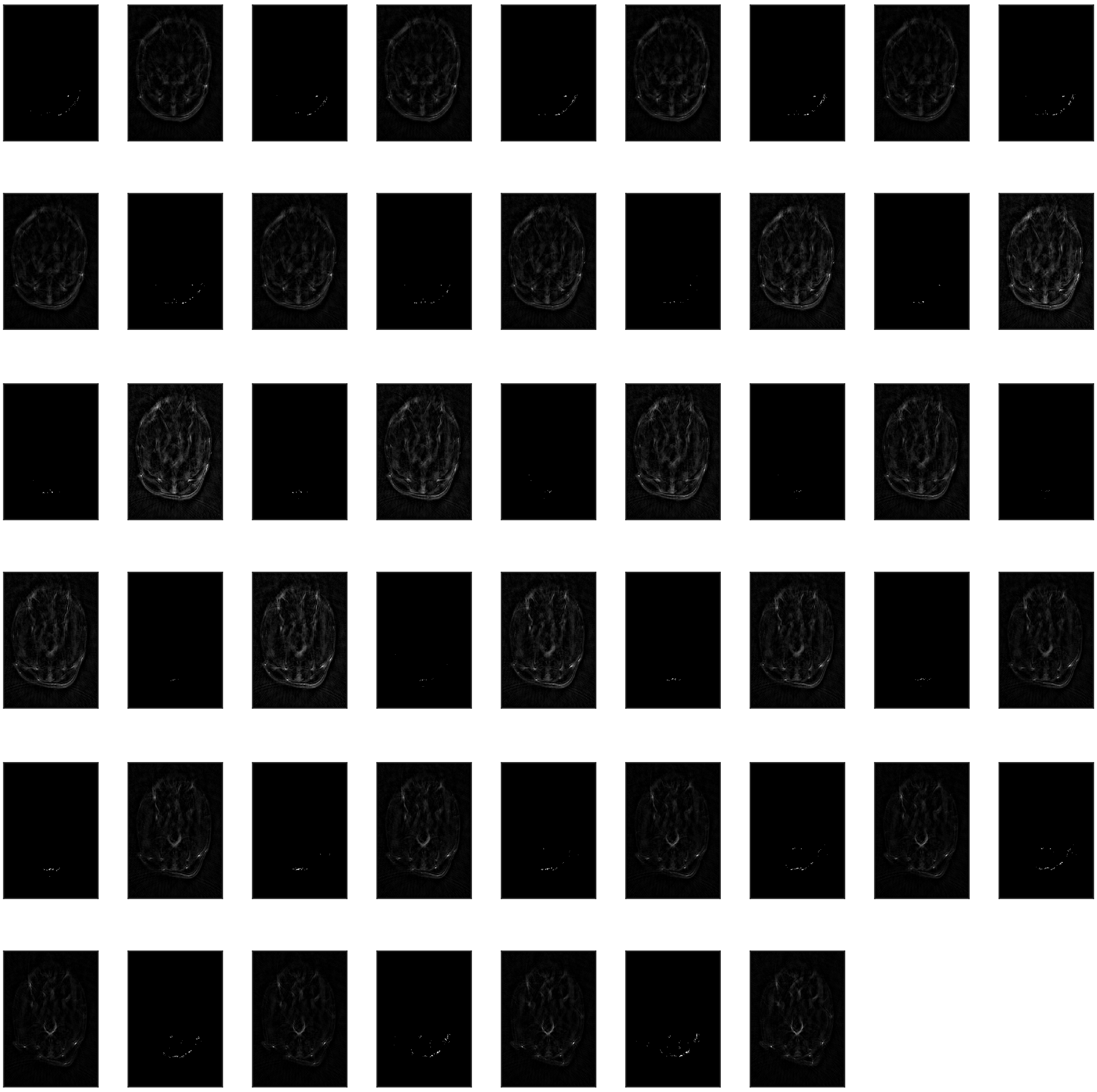

Supplement: Supplementary file 4 — Source Data [file 41467_2019_13041_MOESM4_ESM.zip › Weidenfeld__MSOT_individual_slices_unprocessed.pdf]
